# The dynamics of chemoattractant receptors redistribution in the electrotaxis of 3T3 fibroblasts

**DOI:** 10.1186/s12964-025-02165-4

**Published:** 2025-04-08

**Authors:** Jagoda Gorzkowska, Wiktoria Kozak, Sylwia Bobis-Wozowicz, Ivan Cherepashuk, Zbigniew Madeja, Sławomir Lasota

**Affiliations:** Faculty of Biochemistry, Biophysics and Biotechnology, Department of Cell Biology, Jagiellonian University, Gronostajowa 7, Kraków, 30-387 Poland

**Keywords:** Electrotaxis, Electric field, dcEF, Receptor redistribution, EGFR, Electroosmosis

## Abstract

**Background:**

Electrotaxis, the directed cell movement in direct current electric field (dcEF), is crucial for wound healing and development. We recently proposed a biphasic electrotaxis mechanism, where an initial rapid response is driven by ionic mechanisms, while redistribution of membrane components come into play during prolonged exposure to dcEF.

**Methods:**

To verify this hypothesis, we studied the redistribution dynamics of EGFR, PDGFRα/β, and TGFβR1 in dcEF. For this purpose, we utilized cells transfected with plasmids encoding fluorescently tagged receptors, which were exposed to dcEF in a custom-designed electrotactic chamber. Fluorescent images were captured using wide-field or TIRF microscopy, enabling precise quantitative analysis of receptor redistribution. Additionally, the functional significance of these selected receptors in electrotaxis was evaluated by silencing their expression using an siRNA library.

**Results:**

Although EGFR moved immediately to cathode after dcEF application, maximum distribution asymmetry was reached after 30–40 min. This process was more efficient at higher dcEF intensities, specifically, asymmetry was greater at 3 V/cm compared to 1 V/cm, consistent with the biphasic mechanism observed only under the stronger dcEF. Additionally, redistribution was more effective under alkaline conditions and near the cell base, but decreased when glass was coated with poly-L-lysine, indicating electroosmosis as a key factor. Importantly, EGFR redistribution did not correlate with the rapid reaction of 3T3 cells to dcEF reversal, which occurred within 1–2 min, when receptor orientation was not yet reversed. PDGFRα exhibited similar but less marked cathodal redistribution, while PDGFRβ and TGFβR1 did not redistribute. siRNA knockdown experiments confirmed the importance of EGFR and ErbB4 in the electrotaxis. EGFR’s role was largely ligand-independent, and it had a significant impact on the response of 3T3 cells to dcEF during the first hour of the experiment, but was not involved in the fastest response, which was Kir-dependent.

**Conclusions:**

Our study suggests that EGFR redistribution may play a role in the early stages and partially contribute to the long-term electrotaxis of 3T3 fibroblasts. However, this mechanism alone does not fully explain rapid responses to dcEF orientation changes indicating a more complex, multimodal mechanism of electrotaxis in these cells.

**Supplementary Information:**

The online version contains supplementary material available at 10.1186/s12964-025-02165-4.

## Background

Electrotaxis, the directional movement of cells in response to a direct current electric field (dcEF), is a critical biological process that influences wound healing, embryonic development, and cancer metastasis [1–3]. Most cells exhibiting electrotaxis migrate toward the cathode in a dcEF of physiological magnitude [4]. Physiological dcEFs are formed as a result of transepithelial potential (TEP) disruption, which can occur due to wounding or variability in epithelial conductance [5]. While the ability of cells to undergo electrotaxis is well-documented, the underlying mechanisms facilitating this complex behavior remain only partially understood. The cell membrane, known for its insulating properties, emerges as a critical region for the detection of electric fields, where mechanisms involving either the redistribution of membrane proteins or the activation of ion channels are believed to play central roles [6].

A mechanism based on the redistribution of proteins in the cell membrane was proposed as early as 1977 [7]. The direction of protein redistribution is the result of the combined effects of electrophoresis and electroosmosis acting on proteins [6, 8]. According to calculations, the dynamics of this process during electrophoresis are influenced by the net charge of the molecules at a specific pH, their size, and the characteristics of the cell membrane [8]. In the case of electroosmosis, the opposite direction (than in the case of electrophoresis) of protein accumulation is expected due to the interaction of the electric field with counterions accompanying charged membrane components and their hydration shells [9]. Considering the negative charge (at physiological pH) of most membrane proteins and lipids, primarily associated with conjugated oligosaccharide groups, it is anticipated that a water layer rich in positive ions would pull the proteins towards the cathode [8, 9]. This phenomenon could explain why cathodal accumulation of membrane proteins that are important for cell migration is often observed, even if they carry a negative charge. Confirmation of this phenomenon may be seen in the reversal of this tendency due to the enzymatic removal of negatively charged sugar groups from surface proteins [9].

Regardless of the underlying basis, the result of such a movement of receptor molecules is their accumulation at a specific pole of the cell, leading to polarization of their distribution and ultimately to asymmetric activation of selected signaling cascades [2, 10]. Proponents of this theory repeatedly cite the observable asymmetric distribution of membrane proteins in cells placed in an electric field. For example, the receptor for LDL (low-density lipoprotein) accumulated on the cathodal side of human fibroblasts [11], similar to the EGF (epidermal growth factor) receptor, which was elevated at the same side of Corneal epithelial cells (CEC) [12, 13]. Also reports on the involvement of cathodal accumulation of ConA receptor, and fibronectin receptor, subunit 5α integrin in mouse 3T3 fibroblasts appear significant [14]. On the other hand, other theories suggest that the electrophoresis of specific membrane proteins to the anode is crucial for the migratory responses of fish keratocytes. The involvement of this mechanism was indicated by the dependency of the reaction on the environmental pH, which affected the net charge of proteins and thus the direction of their redistribution in the cell membrane [6]. It is worth noting that although all of these works indicated direction of the membrane molecules redistribution after a specific time since dcEF application, none of them precisely described the dynamics of the process. A molecular flux model has further refined our understanding of these dynamics, predicting the behavior of plasma membrane macromolecules under electric fields [8]. The model aligns well with experimental observations, showing that protein gradients assembled based on electrophoretic and electro-osmotic mobilities can predict the directional accumulation of proteins such as tdTomato-GPI in the plasma membrane of CHO cells. This supports the broader hypothesis that EF-induced protein redistribution is a key mechanism for the detection of electric fields by cells.

From the above information, it is evident that the asymmetric distribution of membrane proteins is a common feature accompanying the directional migration of cells in an electric field. However, few studies on the electrotactic response of cells are dedicated to the dynamics of the observed reaction. Information about the time needed to induce the noticeable reaction is often omitted or not sufficiently highlighted. The dynamics of the process are well characterized by experiments that involve studying responses to the reversal of electric field poles. In cases where this issue has been addressed, it has been repeatedly shown that the reaction occurring in response to an applied electric field happens with very high dynamics. This applies to both normal and cancerous tissue cells, as well as selected lower organisms. For MATLyLu prostate cancer cells, the first morphological changes and directionality of movement were observed within 30 s of stimulus application [15]. A significant dynamic response was also presented for the rapidly migrating blebbing subline of Walker Carcinosarcoma WC256 cells, which react to the electrode switch in an equally short time [16]. In the case of slower migrating cells, i.e. the adherent fraction of mouse bone marrow cells react in less than 1 min after dcEF reversal [17]. Even greater dynamics of the process were observed in studies of lower organisms, such as *Amoeba proteus* [18]. In this case, the response was noticeable within 1 s of the electrode switch. Such observations are useful in the process of searching for the primary mechanism of electric field detection, as they allow for linking to potential detection mechanisms of this field and their localization within the cell.

In light of the above information, it is difficult to explain the occurrence of electrotactic responses on the scale of several minutes, tens or a few seconds, with mechanisms involving only the redistribution of proteins or signaling lipids in the plane of the cell membrane, which usually lasts from a few minutes to even one hour to obtain a significant gradient [7, 12, 13, 19, 20]. While the involvement of these phenomena in controlling and sustaining long-term responses to an electric field cannot be excluded, the primary mechanisms of electric field detection should be sought in alternative phenomena, for example, in activation of specific ion channels and transporters [15, 21–27]. The mechanism of ion channel activation in the electrotaxis process is not fully understood. One of the proposed mechanisms assumes that at the cell edge facing cathode, dcEF could lead to plasma membrane depolarization and further opening of voltage-gated ion channels. At the opposite edge, facing the anode, dcEF would hyperpolarize the cell membrane, modifying the electromotive force for particular ions, especially Ca^2+^ [28]. In our previous research, we observed immediate cathodal migration of 3T3 cells in dcEF, mediated by the activity of inwardly rectifying potassium channels (Kir), specifically Kir4.2. However, prolonged exposure to 3 V/cm dcEF led to cathodal migration even without these channels, suggesting a biphasic response of 3T3 cells to dcEF. The reappearance of directional migration took approximately 1–2 h in 3 V/cm dcEF, while in 1 V/cm dcEF it remained diminished throughout the entire experiment. The subsequent phase of electrotaxis appears to involve a slower mechanism, i.e. redistribution of membrane proteins like EGFR, which was observed after 30 min of exposure to 3 V/cm dcEF and could sustain directional movement over longer periods [29].

Given these findings, our ongoing experiments aim to further dissect the dynamics of membrane protein redistribution immediately after the application of an electric field and its reversal. Detailed elucidation of the process dynamics seems to be crucial to assess if the gradual recovery of directional migration in 3 V/cm dcEF mentioned above (i.e. after Kir channels inhibition) can be explained with this mechanism. For this purpose, we used membrane receptors for chemoattractants fused to fluorescent proteins, which allowed us to monitor the protein redistribution throughout the entire process of the response to dcEF application and reversal. Although our research included the elucidation of a possible mechanism that propels receptor redistribution during the process, the crucial aspect was to verify, if the dynamics of the EGFR accumulation could respond for the second phase of 3T3 fibroblasts electrotaxis and could explain the recovery of the response after the observed cessation.

## Methods

### Cell culture

Mouse 3T3 fibroblasts (ATCC CRL-1658) were maintained in a high-glucose Dulbecco’s Modified Eagle’s Medium (DMEM HG) (Sigma-Aldrich, St. Louis, MO, USA). This medium was enriched with 10% fetal bovine serum (FBS) (Gibco, Waltham, MA, USA), penicillin at a concentration of 100 I.U./ml, and streptomycin at 100 µg/ml, collectively referred to as the ‘complete culture medium’. The cells were incubated under standard conditions of 37 °C with 5% CO_2_. To maintain healthy and proliferative cultures, cells were routinely passaged approximately every three days using a 0.25% trypsin/EDTA solution (Gibco, Waltham, MA, USA). This regular subculturing ensured that cell confluency remained below 80%, thereby preventing overgrowth and ensuring optimal conditions for further experimentation.

### Introduction of genetic constructs to cells

Throughout the research cells were transfected with following plasmids: pCMV-LifeAct-TagGFP2 plasmid (ibidi # 60101); perbB1Y1045F-EGFP which was a gift from Martin Offterdinger (Addgene plasmid # 40267) [30]; pcDNA5FRT-EF-Pdgfralpha-EGFPN and pcDNA5FRT-EF-Pdgfrbeta-EGFPN which were gifts from Lotte Pedersen (Addgene plasmids # 66787 and # 66790 respectively) [31] and mEmerald-ALK5-N-13 and mEmerald-Vinculin-23 which were gifts from Michael Davidson (Addgene plasmids # 62751 and # 54302 respectively). If necessary, the plasmids were multiplied in bacteria using standard procedures, and purified with the Plasmid Midi AX isolation kit (A&A Biotechnology, Gdynia, Poland) according to the manufacturer’s protocol.

The 3T3 fibroblasts were plated in a 24-well plate at a density of 2.5 × 10⁴ cells per well and incubated overnight under standard culture conditions. The following day, transfection was performed following the manufacturer’s instructions, using 0.75 µl of Lipofectamine 3000 reagent (Thermo Fisher Scientific, Waltham, MA, USA) and 0.5 µg of plasmid DNA encoding the desired protein per well. Twenty-four hours after transfection, cells were transferred onto 60 × 35 × 0.2 mm glass slides for use in the electrotaxis chamber as described later.

To obtain 3T3 fibroblasts stably expressing GFP, cells were transduced with a pEGIP lentiviral vector which was a gift from Linzhao Cheng (Addgene plasmid # 26777) [32] according to the protocol described before [29], followed with antibiotic selection using 10 µg/ml puromycin (Sigma-Aldrich, St. Louis, MO, USA).

### Cell seeding and application of electric field

Electrotaxis was examined with an electrotactic setup described before [29, 33, 34]. Briefly, the day before the planned experiment, cells were seeded at a density of 10⁴ cells in 400 µl of complete culture medium on a previously sterilized 60 × 35 × 0.2 mm glass slide placed in a sterile dish, resulting in a final cell density of approximately 2.5 × 10³ cells/cm². The dish was then transferred to a humidity chamber and placed in an incubator.

On the day of the experiment, two 60 × 10 × 0.2 mm glass slides were taped along the longer edges of the another 60 × 35 × 0.2 mm glass slide using double-sided adhesive tape (Tesa SE, Hamburg, Germany, # 64621). This construct was then attached to the slide with the previously seeded cells and filled with additionally buffered culture medium. To prevent pH fluctuations during the experiment, a powder DMEM HG culture medium without NaHCO₃ buffer (Sigma-Aldrich, St. Louis, MO, USA) with 10% FBS and antibiotics (penicillin and streptomycin) was supplemented with HEPES buffer (for pH 7 and 8) or MES buffer (for pH = 6.0) (both Sigma-Aldrich) to a final concentration of 15 mM, and pH was adjusted. The glass observation chamber containing the cells was transferred to a special PVC electrotaxis chamber and sealed with silicone paste. The internal reservoirs were filled with 7 ml of pH-adjusted medium each, and the silver measuring electrodes served for monitoring the potential difference across the ends of the glass chamber using a voltmeter during the experiment. The external reservoirs containing the silver chloride electrodes (Ag|AgCl, 7.5 cm²) were filled with a PBS solution. The internal and external compartments were connected with glass salt bridges filled with 2% agar in 0.5 M KCl. After placing the electrotaxis chamber under the microscope, it was connected to a power supply. In this setup, the cathode was negatively charged, while the anode carried a positive charge. Consequently, the ion current, defined by cation migration, flowed from the anode toward the cathode within the chamber. The wiring setup allowed for quick reversal of the electric field direction if needed [33]. Cells were stimulated with an electric field of 1 and 3 V/cm.

### Visualization of membrane proteins redistribution and dynamics of electrotaxis

Imaging was performed 48–72 h after transient transfection or 24 h after reseeding of GFP-expressing cells, using a Leica DMI8 inverted fluorescence microscope equipped with a DFC7000 GT monochromatic CCD camera, a dry HCX APO U-V-I 40×/0.75 objective, a GFP-optimized filter set including QUAD filter cube and fast filter wheels at FITC position (all from Leica, Wetzlar, Germany), and a CoolLED pE-4000 LED illuminator (CoolLED Ltd., Andover, UK) set at 490 nm diode. The microscope was working under the control of LAS X 3.7 software (Leica, Wetzlar, Germany). Throughout the entire experiment, cells were kept at 37 °C maintained with an environmental chamber and temperature controller (PeCon GmbH, Erbach, Germany). Fluorescent cell images were captured every 30 s across multiple fields of view. Precise maintenance of image focus was achieved using the AFC (Adaptive Focus Control) module. Recording was done for 10 min before and 120 min after applying the electric field (after one hour of cell stimulation with the cathode of dcEF on the right, the negative and positive electrodes were switched by manually reversing the wires in the power supply). For subsequent quantitative analysis, all films regarding particular protein were recorded with consistent parameters, including excitation light intensity, pixel binning, electronic gain, and exposure time. Images were subsequently processed using ImageJ Fiji software (National Institutes of Health, Bethesda, MD, USA), including shading correction, background subtraction and the creation of plot profiles depicting pixel intensities along designated lines for specified time-points. Fluorescence values were normalized to the brightest pixel along the line.

### TIRF microscopy and poly-L-lysine coating

To more accurately visualize the redistribution of the EGF receptor in the plasma membrane adjacent to the substrate, total internal reflection fluorescence microscopy (TIRFM) was employed. The selective excitation of fluorophores within the plasma membrane, combined with high spatial resolution and low background noise, makes this method highly sensitive for studying processes occurring in the cell membrane. TIRFM was performed using an automated Leica DMI6000B fluorescence microscope equipped with an HC PL APO 100×/1.47 OIL objective, a 488 nm diode laser as the light source (all from Leica, Wetzlar), and Type 37LDF immersion oil (Cargille, 16240). The penetration depth of the evanescent wave was set to 110 nm from the glass/culture medium interface. Cells were imaged for 65 min (5 min under no dcEF conditions and the next 60 min under 3 V/cm dcEF, with electrodes reversal after 30 min).

To reverse the electroosmotic flow on the substrate, a solution of poly-L-lysine (0.1 mg/ml) (Sigma-Aldrich) was spread onto a 60 × 35 × 0.2 mm glass slide placed in a sterile dish and left for 5 min. The excess reagent was removed with a pipette and the surface was rinsed three times with sterile water. The slide was then allowed to dry in a laminar flow hood. Cells were seeded as previously described.

### Analysis of protein distribution

**Fig. 1:**
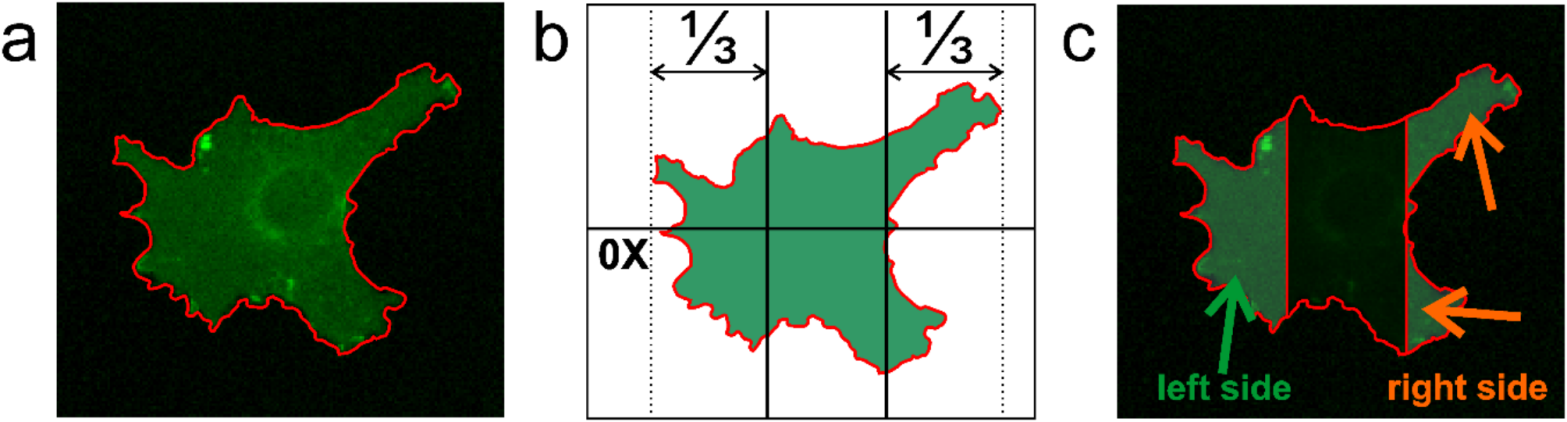
Schematic representation of cell sectioning for quantitative analysis of fluorescence intensity and cell region area changes. (**a**) Fluorescence images were automatically segmented, and (**b**) cells were sectioned into three equal regions along the 0X axis, parallel to the direction of dcEF lines (if present), based on the farthest points to the right and left. Each region comprised one-third of the line segment spanning from the farthest points projected onto the 0X axis. (**c**) The right and left sides of the cell were selected for further analysis, while the central region was excluded. For fluorescence intensity measurements, cell sectioning was performed at each time step, whereas for area analysis, sectioning was only executed at time t = 0 min (denoting dcEF application or reversal)

Quantitative analysis of proteins distribution in cell membrane was conducted using a dedicated macro operating within the Fiji ImageJ software [35]. After optimizing the parameters, this macro automatically thresholds the fluorescence intensity and delineates the cell outline from the field of view (Fig. 1a), which is then transferred to the raw image. At each time point, the background intensity is measured and subtracted from the cell signal. Subsequently, for each time point, the cell is sectioned along an axis parallel to the direction of the electric field (0X) into three equal parts (Fig. 1b), based on which the fluorescence intensity is measured (Fig. 1c). The obtained values corresponding to each cell region (i.e., the right or left side, as measurements were not taken for the central region) were normalized to the fluorescence intensity of the entire cell. This procedure allowed for the comparison of cells with varying levels of fluorescence intensity. Additionally to ensure reliable fluorescence measurements, we selected only cells with medium or high fluorescence intensity, and a regular, symmetric shape at the start of the experiment. This minimized errors from uneven sectioning, which could lead to biased fluorescence intensity measurements in smaller regions. Graphs were prepared using GraphPad Prism 8.0.1 software (GraphPad Software, Boston, MA, USA).

### Analysis and visualization of electrotaxis dynamics

To analyze the dynamics of electrotaxis, the previously mentioned macro was modified to partition the cell into three sections, setting the sectioning lines at the time point corresponding to the application or reversal of the dcEF, analogically to analyses conducted previously [17, 29]. This cell sectioning remained stable throughout the entire analyzed time window (in contrast to the analysis of receptors redistribution, which was performed dynamically for each image). By tracking the increase or decrease in the area of the right and left sides of the cell during the periods before and after these events relative to the areas measured at the time of cell sectioning (normalized to 1), we determined the precise moment of the cell’s response to the dcEF. This analysis was carried out on 3T3 fibroblasts that exhibited stable expression of GFP localized in the cytoplasm, obtained as described before, or on cells expressing EGFR-GFP.

The dynamics of the response can also be visually assessed in the supplementary video (Additional file 2) and extracted frames (Fig. 4), where a series of overlaid images is presented. Red corresponds to the moment of application of dcEF or its reversal, while the green channel changes dynamically with each time point. As a result, regions newly covered by the cell appear green, areas retracted are red, and regions showing no change over the observed time period appear yellow.

### Screening for genes of membrane receptors and analysis of single cell migration

In order to investigate the role of specific membrane receptors in cellular electrotaxis, we utilized 16 different sets of siRNA constructs sourced from the Mouse ON-TARGETplus siRNA library - Tyrosine Kinases, SMARTpool (Dharmacon, Lafayette, CO, USA; Cat No. G-113105). The siRNA solutions were prepared in accordance with the manufacturer’s instructions, resulting in a stock solution concentration of 5 µM in 20 µl of 1× siRNA buffer (Dharmacon). 3T3 fibroblasts were cultured in 24-well plates at a density of 3 × 10⁴ cells per well in DMEM HG medium supplemented with 10% FBS but without antibiotics. After 24 h, cells were transfected using Dharmafect I reagent (Dharmacon) as per the manufacturer’s guidelines, with 1 µl of transfection reagent and 2.5 µl of the prepared siRNA stock solution added to each well. Efficiency of this siRNA formulation was confirmed using two independent methods (Fig. S1), namely knockdown of GFP in stably transduced cells (with GFP Duplex I siRNA, Dharmacon) and GAPDH knockdown (with ON-TARGETplus GAPD Control siRNA, Dharmacon) verified by Western blot.

Following a 24-hour transfection period, the cells were transferred to the basal glass slide of the electrotactic chamber (measuring 60 × 35 × 0.2 mm) using a 9-well PDMS stencil (Alvéole, Paris, France) placed centrally, seeding at a density of 400 cells per 9 mm² area in 10 µl of complete culture medium. The cells were maintained in a humidity-controlled environment until the experiment commenced, which was 48 h post-transfection. At the time of the experiment, the stencil was removed, and the electrotactic chamber was assembled according to the protocol described previously. This modified setup enabled simultaneous time-lapse imaging of eight different cell types with specific gene knockdowns, alongside control cells transfected with nontargeting siRNA. The imaging was performed using a fully automated Leica DMi8 microscope, equipped with an environmental chamber, an MC170-HD CMOS camera, and an HC FL PLAN 10×/0.25 DRY objective, all operated via LAS X 3.7 software (Leica, Wetzlar, Germany). Images were captured from three distinct fields of view for each condition, taken sequentially every 5 min over a period of 4 h and 30 min. After the initial 30 min of imaging, a direct current electric field (dcEF) of 3 V/cm was applied with the cathode on the right. Cell migration analysis was carried out on 30 randomly selected cells per condition.

Single cell migration analysis was conducted as previously described [16, 29, 33, 36] using Hiro 1.0.0.4 software (W.Czapla, Krakow, Poland) which allowed us to construct cell migration trajectories based on manually identified cell centroids. To generate circular diagrams, the trajectory of each cell was adjusted so that its starting point coincided with the origin of the coordinate system. The directionality of cell migration was evaluated using the average directional cosine γ, which measures the angle between the line connecting the initial trajectory point to subsequent cell positions and the X-axis (aligned parallel to the dcEF vector). A γ value close to 1 indicates that the cells are migrating toward the cathode, while a γ value near − 1 signifies movement toward the anode. An average γ value approaching 0 suggests random movement of cells in the population without a preferred direction. Directional cosine γ values were then plotted to visualize the migration patterns, and statistical analysis was performed to determine the significance of any differences in migration directionality compared to the control group (treated with non-targeting siRNA).

### Analysis of electrotaxis in serum-free conditions

Cells were seeded onto glass slides as described previously (see Cell seeding and application of electric field). However, instead of complete culture medium with FBS, the assembled observation chamber was filled with serum-free medium prepared analogously (pH adjusted to 7). Where indicated, this medium was supplemented with recombinant mouse EGF (Sigma-Aldrich, SRP3196) at final concentrations of 1 or 5 ng/ml, barium chloride (BaCl₂, Sigma-Aldrich, 500 µM) to inhibit Kir channels, or the EGFR inhibitor AG1478 (Sigma-Aldrich, 3 µM).

Cell migration under these serum-free conditions was recorded using a Leica DMi8 inverted microscope equipped with N PLAN L 20×/0.35 DRY objective and a DFC7000GT camera in DIC (Differential Interference Contrast) mode, as described previously. Owing to the increased sensitivity of cells in serum-free medium, the total recording time was shortened to 3 h and 30 min; with the dcEF applied after the initial 30 min. Migration analysis was carried out on 50 randomly selected cells per condition, according to the methods detailed earlier (see the previous section).

### Statistical analysis

To evaluate the statistical significance of differences in cell migration directionality, the non-parametric Mann–Whitney U test was employed, as this parameter did not follow a normal distribution (as verified by the Shapiro-Wilk test). In experiments involving multiple conditions, where variance differences between groups were significant (confirmed by Levene’s test), this test was further justified by its robustness to heterogeneity in variance. All statistical analyses were performed using GraphPad Prism version 8.0.1 (GraphPad Software, Boston, MA, USA), except for Levene’s test, which was performed online (StatsKingdom.com), with a significance level established at *p* < 0.05.

## Results

### EGFR redistribution as a component of the biphasic mechanism of the electrotaxis of 3T3 fibroblasts

**Fig. 2:**
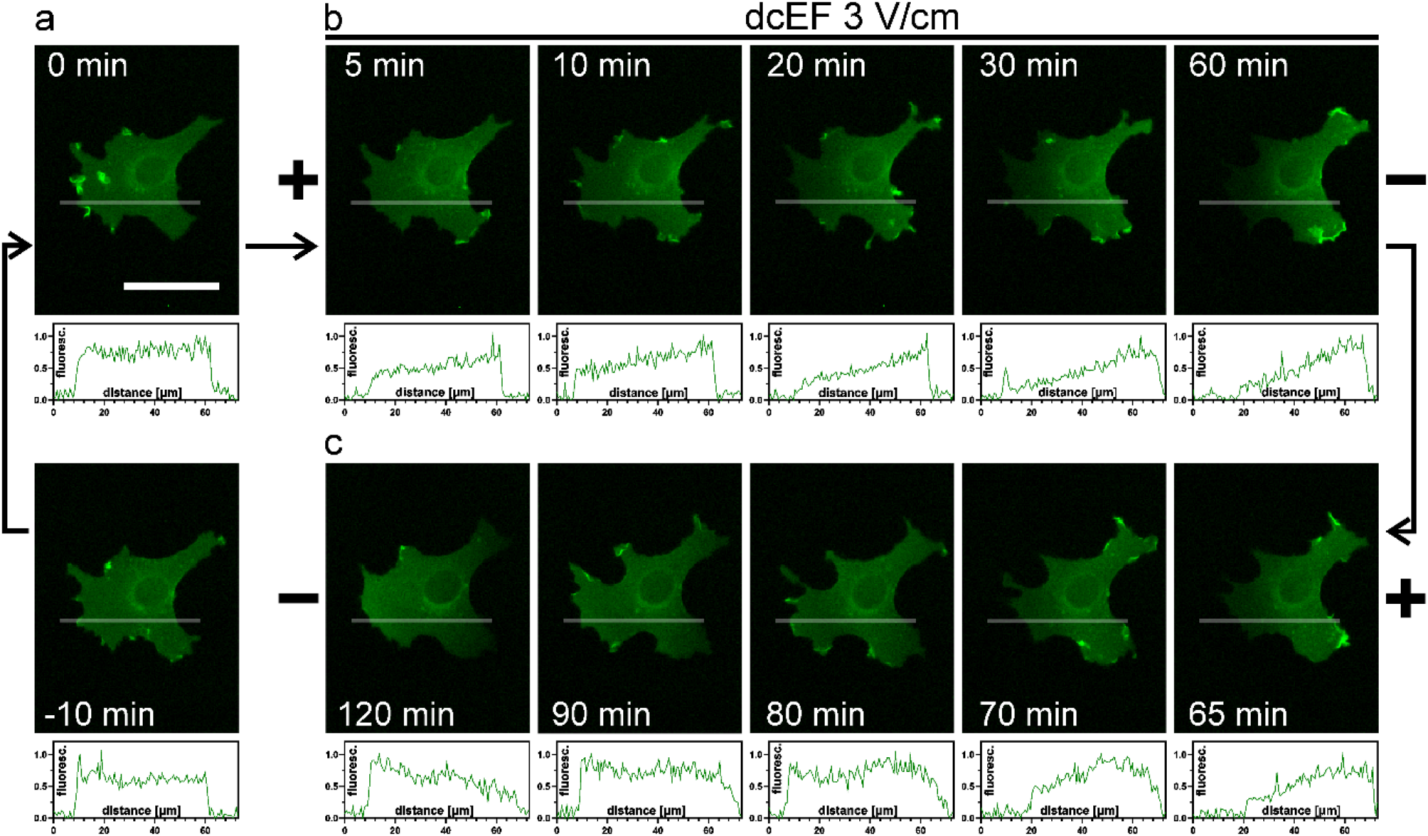
Visualization of EGFR-GFP redistribution in response to dcEF. 3T3 fibroblasts transfected with a plasmid encoding EGFR-GFP were observed using fluorescence microscopy before and during the application of a dcEF (3 V/cm). The succession of presented images is annotated with arrows. Initially, a 10-minute recording captured cells in isotropic conditions (**a**) (10 min before EF application (-10 min), at the moment of EF application (0 min)). Subsequently, the dcEF was applied with the cathode placed on the right side for 60 min (**b**). Following this, the dcEF polarity was reversed by replacing the electrodes and placing the cathode on the left side (**c**). The line profiles illustrate the relative distribution of fluorescence within the cell, normalized to the brightest pixel along the line. A scale bar of 50 μm is applicable to all images within the figure

Given our previous proposal of a biphasic mechanism for the electrotaxis of 3T3 fibroblasts [29], where the activity of the ion channels, particularly that of the Kir4.2 channel, accounts for the initial rapid response to dcEF, and the redistribution of membrane receptors stabilizes the second phase, we now aim to determine the exact timing of EGFR accumulation within the cell membrane. Based on our hypothesis, it would be expected that substantial redistribution of crucial membrane proteins would occur around the end of first hour of dcEF application, as between 1 and 2 h directionality of migration in 3 V/cm dcEF gradually recovered to the control values. Previously, we demonstrated via immunocytochemistry that EGFR accumulates on the cathode side after prolonged exposure to dcEF [29]. In this study, we further investigate this process using 3T3 cells expressing EGFR tagged with a fluorescent protein (EGFR-GFP). Using fluorescence microscopy with a 30-second time resolution, we captured the dynamics of EGFR redistribution before, during dcEF application, and after its polarity reversal. Figure 2 and Supplementary Movie 1 (Additional file 1) illustrate these observations, showing the changes in EGFR-GFP distribution over time in response to the electric field.

**Fig. 3:**
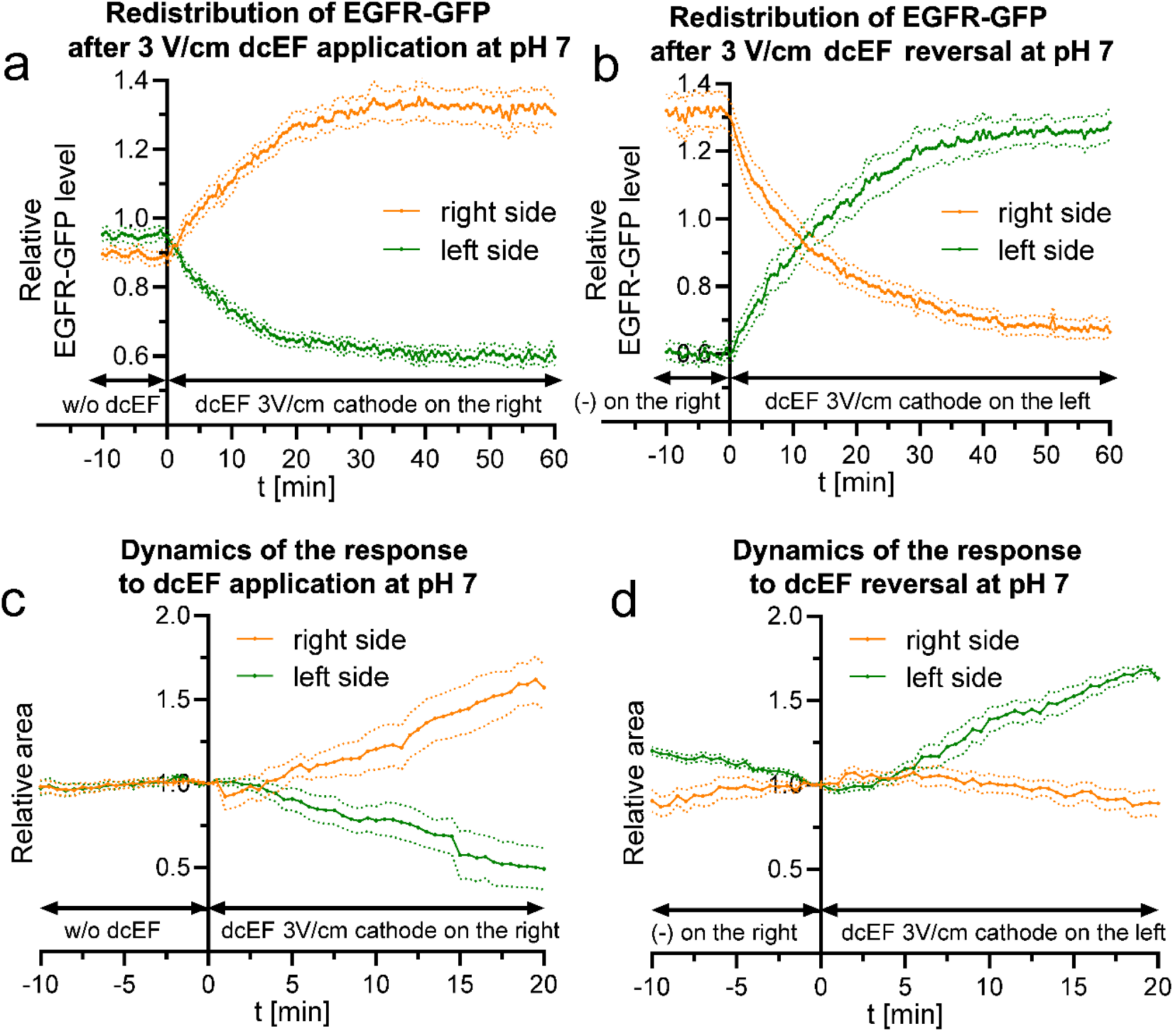
Quantitative analysis of EGFR-GFP redistribution and dynamics of 3T3 fibroblasts electrotaxis following dcEF application and reversal (**a**) - redistribution of the fluorescent protein after the application of dcEF (3 V/cm) at the 0-minute time point, with the cathode placed on the right side of the field of view. Fluorescence intensities were measured every 30 s for both the right and left sides of the cell (designated at each time point). The values were subsequently normalized by dividing them with the average fluorescence intensity of the entire cell, and are presented as orange and green lines, respectively. (**b**) - redistribution of the fluorescent protein following the reversal of the dcEF (3 V/cm) polarity at the 0-minute time point, achieved by replacing the electrodes and transferring the cathode to the left side of the field of view. The graph was constructed as previously (**a**). The graphs (**a**, **b**) represent average values (± SEM) for *n* = 17 cells from 4 independent experiments; (**c**) - variations in the areas of the cell regions after the application of a dcEF (3 V/cm) at the 0-minute time point, with the cathode placed on the right side of the field of view. The areas of fluorescent cell regions were measured every 30 s for both the right and left sides of the cell (designated at time point 0 min). These values were subsequently normalized to 1 at the time of sectioning and are presented as orange and green lines, respectively. (**d**) - variations in cell region areas after reversing the dcEF polarity at the 0-minute time point, achieved by replacing the electrodes and transferring the cathode to the left side of the field of view. The graph was constructed using the same methodology as in (**c**). The graphs (**c**, **d**) represent average values (± SEM) for *n* = 6 cells from 3 independent experiments. Throughout these experiments, the culture medium pH was maintained consistently at 7

The observed distribution of the EGFR-GFP fusion protein was initially uniform across the cell membrane (Fig. 2a). Upon application of a 3 V/cm dcEF, a rapid accumulation of the protein on the cathode-facing side was detected, as evidenced by an increase in fluorescence signal (Fig. 2b). When the polarity of the dcEF was reversed, a gradual migration of the protein toward the new cathode was observed (Fig. 2c). These results confirm that EGFR redistribution occurs swiftly in response to the electric field and that this redistribution is reversible with changes in field polarity, although the dynamics of EGFR redistribution proved to be much faster than anticipated under the proposed model. Consequently, we conducted a detailed quantitative analysis in numerous cells, following the described methodology. The results, which illustrate the changes in the fluorescence signal on the cathode and anode sides, are presented in Fig. 3a and b. Additionally, we compared EGFR redistribution with 3T3 fibroblasts electrotaxis dynamics (Fig. 3c and d).

In line with the earlier visual assessment, the accumulation of EGFR on the cathode side begins immediately after the application of the dcEF, accompanied by a symmetrical decrease on the anode side (Fig. 3a). The final excess reaches up to 40% of the total cellular fluorescence, stabilizing between 30 and 40 min after dcEF application. Such rapid receptor redistribution could solely explain the dynamics of the electrotactic response observed in Fig. 3c, where an increase in cell surface area on the cathode side becomes evident around 4 min after applying the 3 V/cm dcEF.

However, it is important to note the graph characterizing receptor redistribution after reversing the electric field (Fig. 3b). The key information here is the time required for the polarity reversal of the receptor distribution as it takes up to 13.85 ± 2.23 min for the receptor to predominate on the ‘new cathode’ side. At the same time, the graph of cell movement dynamics in this setup (Fig. 3d) shows the shift of protrusive activity to the ‘new cathode’ side within 1–2 min of polarity reversal, so much earlier than substantial EGFR redistribution occurs.

**Fig. 4:**
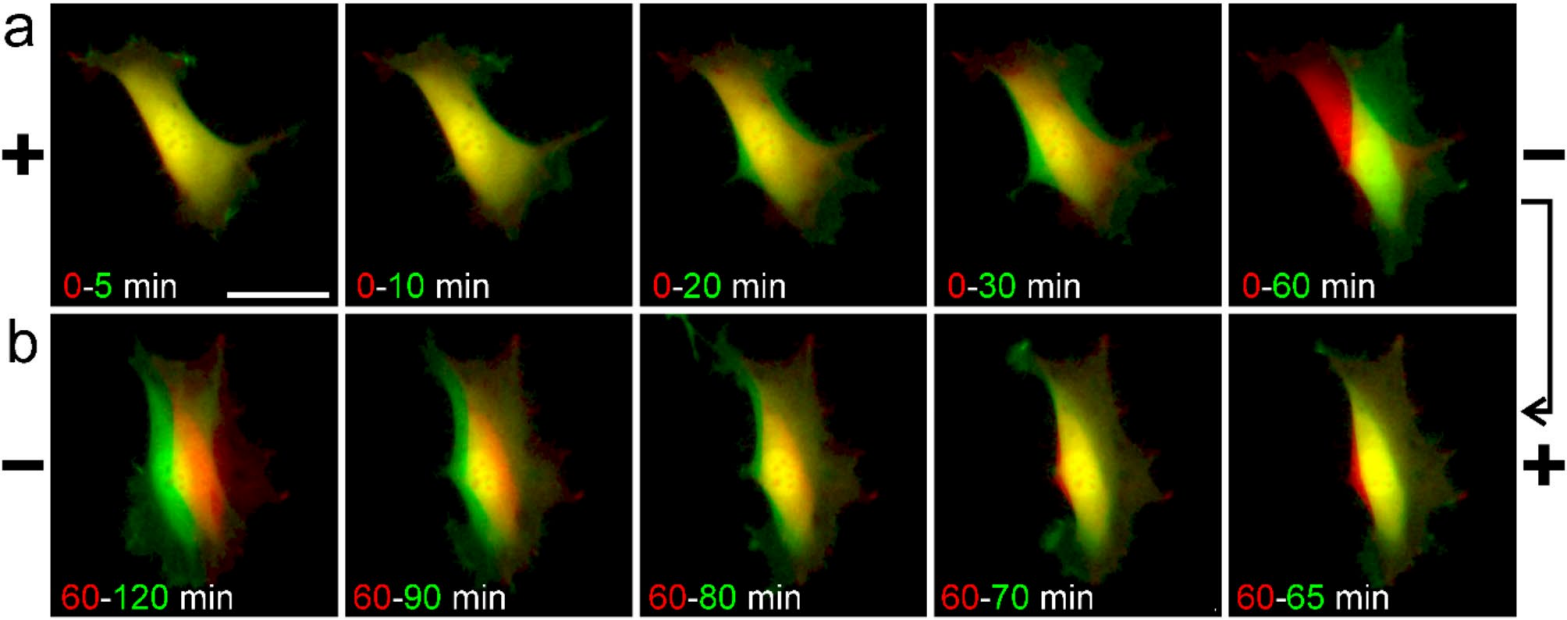
Visualization of 3T3 fibroblasts electrotaxis dynamics. Cells expressing GFP were recorded with fluorescence microscopy after the application **(a)** and reversal **(b)** of a 3 V/cm dcEF. The green image of the cell at the specified time point was overlayed with the red image of the cell at the reference time point, where the dcEF of 3 V/cm was applied **(a)** or reversed **(b)**. As a result, green color denotes a region covered, while red color denotes a region released by the cell between the reference time and the specified time. The scale bar is 50 μm. The complete image series recorded at 30-second intervals can be found in Supplementary Movie 2 (Additional file 2)

Such a high dynamics of electrotaxis is clearly visible in the microscope images, where cells expressing GFP are shown (Fig. 4, Additional file 2).

Rapid protrusion occurs on the cathode side within the first minutes after dcEF application (Fig. 4a) and reversal (Fig. 4b), which is shown as the newly covered green area. The physiological nature of the response is further emphasized by the dynamics of the actin cytoskeleton and focal contacts (Supplementary Movies 3 and 4 – Additional files 3 and 4, respectively). This confirms that the new protrusions are based on rapid actin polymerization and, after a slight delay, they attach to the cell substratum with newly formed focal contacts. It is worth mentioning that even the latter occurs on the ‘new cathode’ side within the first 10 minutes after dcEF reversal. At the level of the cytoskeleton and focal adhesions, it is clear that cell retraction on the ‘new anode’ side also begins within the first few minutes after dcEF reversal.

**Fig. 5:**
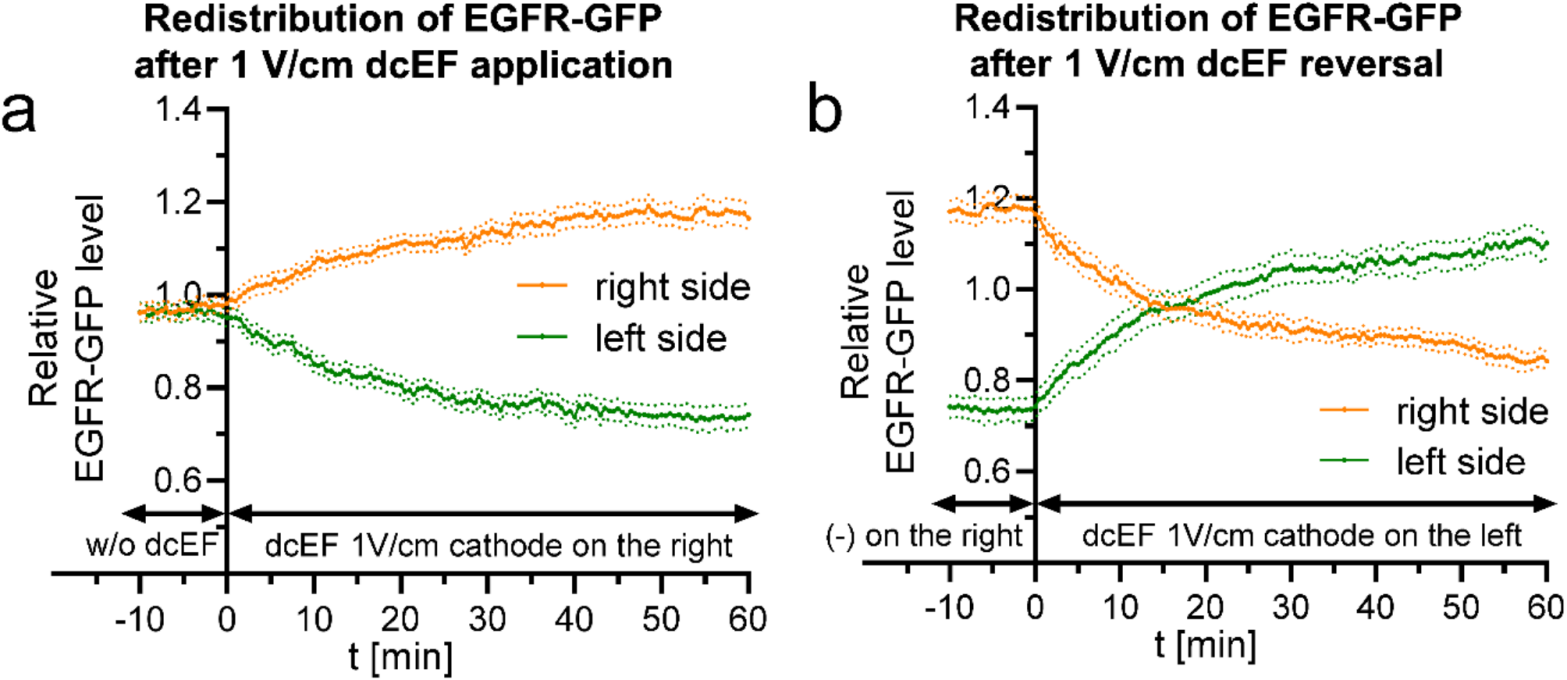
Quantitative analysis of EGFR-GFP redistribution following 1 V/cm dcEF application and reversal **(a)** - redistribution of the fluorescent protein after the application of 1 V/cm dcEF at the 0-minute time point, with the cathode placed on the right side of the field of view. Fluorescence intensities were measured every 30 s for both the right and left sides of the cell (designated at each time point). The values were subsequently normalized by dividing them with the average fluorescence intensity of the entire cell, and are presented as orange and green lines, respectively. **(b)** - redistribution of the fluorescent protein following the reversal of the dcEF (1 V/cm) polarity at the 0-minute time point, achieved by replacing the electrodes and transferring the cathode to the left side of the field of view. The graph was constructed as previously **(a)**. The culture medium pH was maintained at 7. The graphs represent average values (± SEM) for *n* = 16 cells from 3 independent experiments

In our previous studies mentioned [29], the complete recovery of directionality in the setup with inhibited or silenced Kir4.2 occurred at 3 V/cm dcEF but not at 1 V/cm, which (assuming the crucial role of examined mechanism) suggests that only strong enough dcEF is able to effectively drive membrane proteins redistribution. Therefore, we also decided to characterize the EGFR redistribution at this lower field intensity (Fig. 5).

In this case, redistribution also occurred, but the extreme shift reached only about 20% relative to the average signal for the cell and was achieved between 40 and 50 min after the application of the electric field. The time required to reverse the equilibrium after changing the field polarity was more than 15 min in this instance.

In light of our previous observations and the proposed biphasic model, this result may suggest that a sufficiently large disparity in membrane receptor distribution, such as EGFR, is necessary to direct the migration of 3T3 fibroblasts. Therefore, this mechanism alone cannot account for the very rapid cellular response to the electric field, but is well-suited to explain the stability of the response over a longer time scale, when in fact electrotaxis plays its role i.e. in the process of wound healing, especially at higher electric field intensities.

### Verification of the EGFR redistribution driving force

**Fig. 6:**
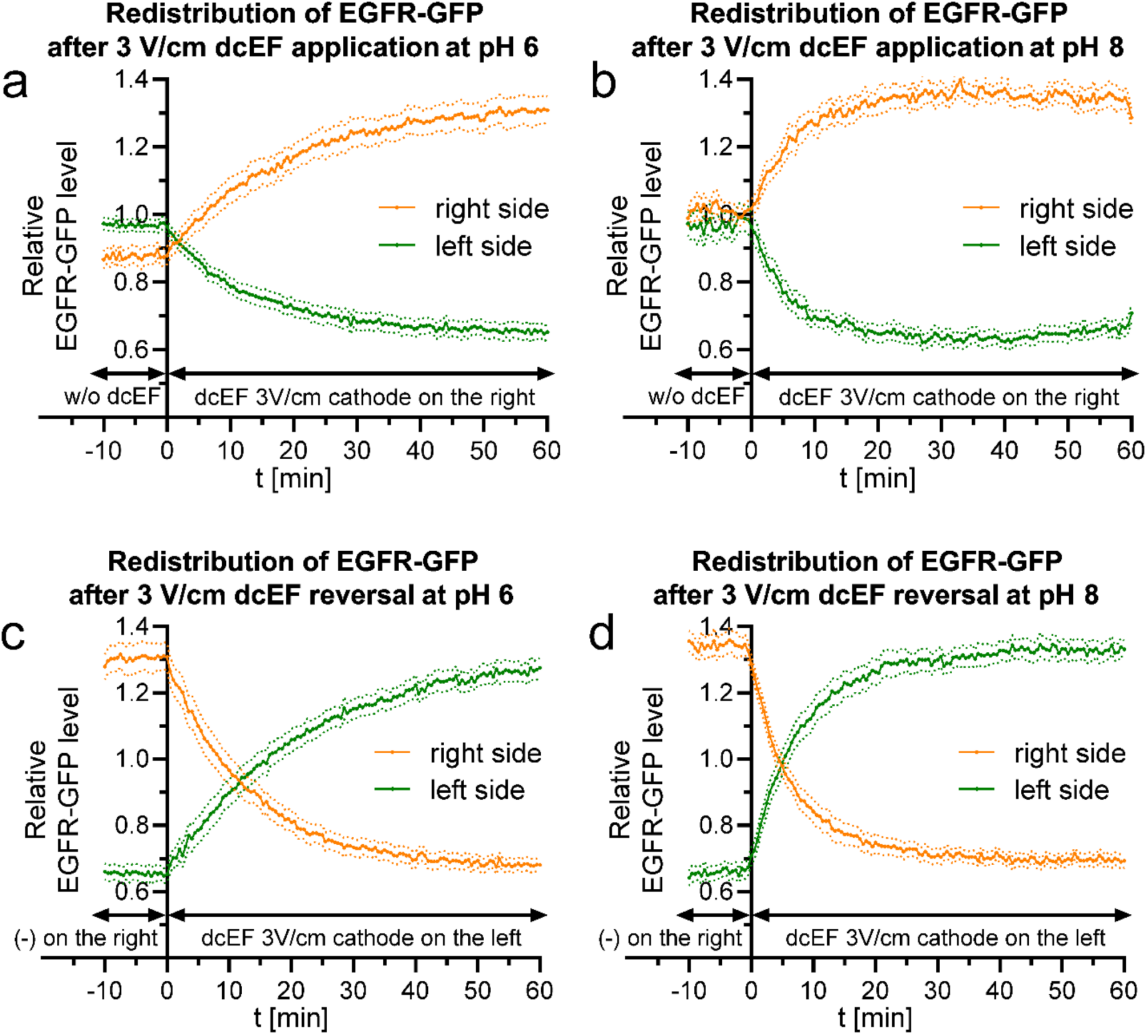
Impact of culture medium pH on EGFR-GFP redistribution **(a**,** b)** - redistribution of the fluorescent protein following the application of dcEF (3 V/cm) at the 0-minute time point, with cathode placed on the right side of the field of view. Fluorescence intensities were measured every 30 s for both the right and left sides of the cell (designated at each time point). The values were then normalized by dividing them by the average fluorescence intensity of the entire cell, and are depicted as orange and green lines, respectively. **(c**,** d)** - redistribution of the fluorescent protein after reversing the dcEF (3 V/cm) polarity at the 0-minute time point, achieved by replacing the electrodes and transferring the cathode to the left side of the field of view. The graphs were constructed following the same methodology as in **(a)** and **(b)**. In both sets of experiments, the culture medium pH was adjusted and maintained at either 6 **(a**,** c)** or 8 **(b**,** d).** The graphs represent average values (± SEM) for *n* = 20 cells (pH 6) and *n* = 15 cells (pH 8), each group derived from 3 independent experiments

Recognizing the presence and possible role of the redistribution-based mechanism in long-term electrotaxis, we investigated the primary forces responsible for receptor accumulation over time. As mentioned in the introduction, the main driving forces are expected to be electrophoresis or electroosmosis, which should respond differently to changes in the extracellular pH. Therefore, we extended our study to investigate the dynamics of this process at pH 6 and pH 8, during both – application and reversal of the dcEF 3 V/cm (Fig. 6).

We can see that acidification of the environment decreased the observed dynamics of EGFR-GFP redistribution (Fig. 6a) as in contrast to the neutral pH (Fig. 3a), here the signal did not stabilize during the 60 min of dcEF application. On the contrary, an alkalization of culture medium considerably increased the dynamics leading to signal stabilization within 20–30 min of 3 V/cm dcEF application (Fig. 6b). This is even more evident in the case of the time required to reverse equilibrium after the dcEF reversal (Fig. 6d), which was reduced to 6.83 ± 1.39 min at pH 8 (compared to 13.85 ± 2.23 min at pH 7). At elevated pH levels, an increase in the net negative charge on the cell surface is expected, leading to a higher concentration of positive counterions and thus enhancing the electroosmotic process towards the cathode. This observation indicates that electroosmosis is the primary driving force behind the redistribution of EGFR toward the cathode. In contrast, if the electrophoresis was the driving force of EGFR redistribution, assuming its positive charge at pH 7 (a necessary assumption due to cathodal accumulation), further alkalization to pH 8 would reduce the efficacy of the process, due to reduction of protein charge. Oppositely, the dynamics should have been elevated at pH 6.

**Fig. 7:**
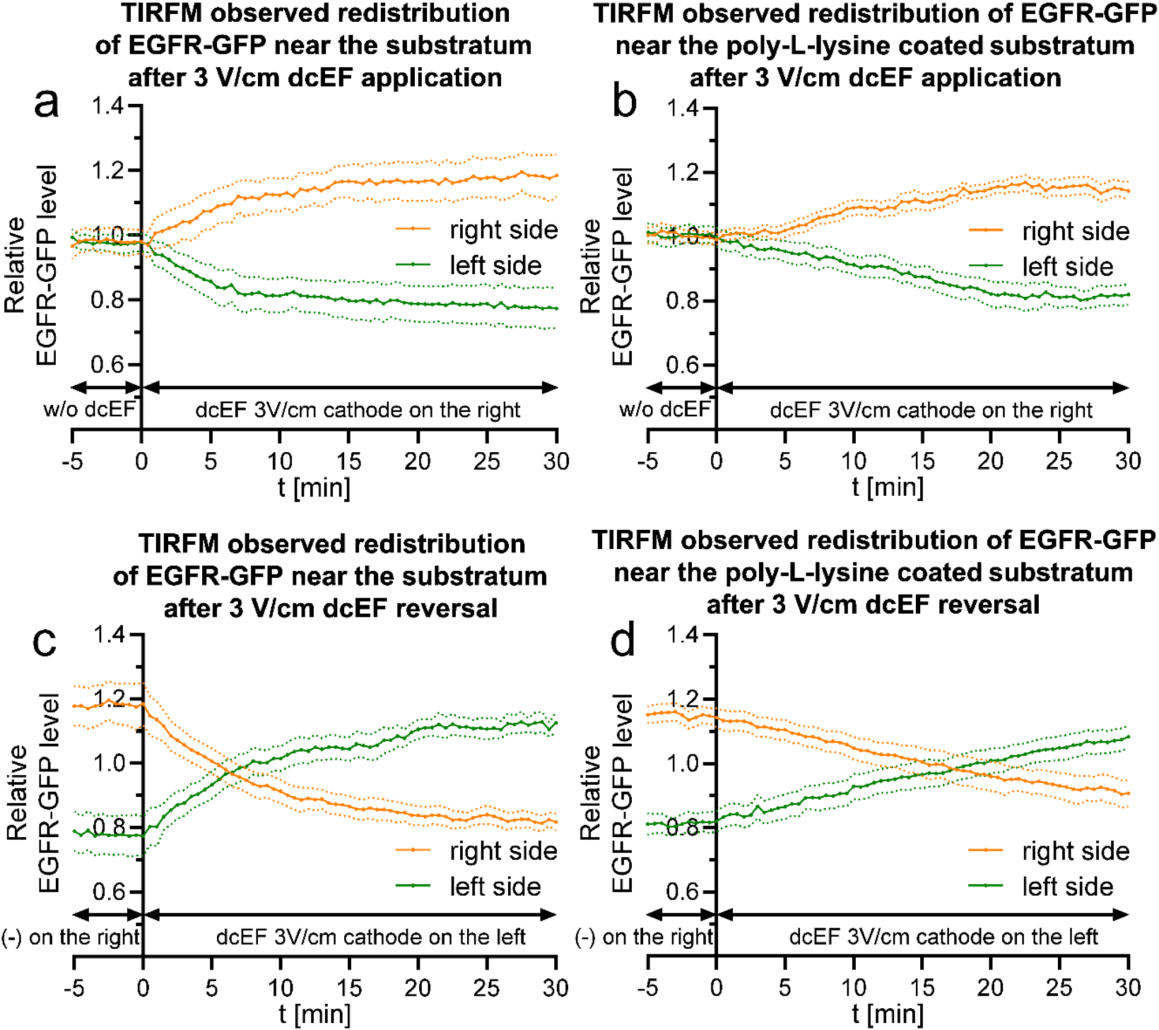
Quantitative analysis of TIRFM observed EGFR-GFP redistribution near the substratum following dcEF application and reversal - the involvement of electroosmosis near the substratum (**a**, **b**) - redistribution of the fluorescent protein after the application of dcEF: (**a**) - glass substratum; (**b**) - coated with poly-L-lysine (0.1 mg/ml). At the 0-minute time point, the cathode was placed on the right side of the field of view. Fluorescence intensities were measured every 30 s for both the right and left sides of the cell (designated at each time point). The values were subsequently normalized by dividing them by the average fluorescence intensity of the entire cell, and are presented as orange and green lines, respectively. (**c**, **d**) - redistribution of the fluorescent protein following the reversal of the dcEF polarity: (**c**) - glass substratum; (**d**) - coated with poly-L-lysine (0.1 mg/ml). At the 0-minute time point electrodes were replaced, transferring the cathode to the left side of the field of view. The graphs were constructed as previously (**a**, **b**). The graphs show average values (± SEM) for *n* = 6 cells on glass substratum and *n* = 6 cells on poly-L-lysine coated surfaces, each group derived from 5 independent experiments

As the electroosmosis is the primary driving force, EGFR redistribution should be particularly pronounced at the cell surface/glass interface, where it is driven by two components: the negative zeta potential of the cell membrane and the negative charge of the glass surface on which the cell migrates. In the next experiment we used TIRF microscopy to characterize the redistribution of EGFR in the membrane adjacent to the substrate (Fig. 7a and c). Additionally, we modified the glass surface charge by coating it with poly-L-lysine (Fig. 7b and d).

Indeed, it was demonstrated that redistribution in this region was particularly pronounced, as it occurred very dynamically, with equilibrium reversal observed just 6.5 min after reversing the polarity of the 3 V/cm dcEF (Fig. 7c). Reversing the charge on the substrate surface did not completely eliminate redistribution in this region but reduced its dynamics to a level even lower than that observed across the entire cell (Fig. 7d), likely driven by electroosmosis at the immediate surface of the cell membrane.

### PDGF receptors redistribution during the electrotaxis of 3T3 fibroblasts

To further validate that our observed results indeed reflect the redistribution of membrane proteins due to the influence of dcEF, we investigated the redistribution of two additional receptors: PDGFRα and PDGFRβ. While PDGF was characterized in the past as a chemoattractant for fibroblasts, based on The Human Protein Atlas, PDGFRα is known to be localized in the cell membrane, while PDGFRβ is expected to localize intracellularly [37]. Such a distribution was confirmed in our cells through immunofluorescence staining (Fig. S2a and b), where PDGFRβ exhibited notably lower localization at the plasma membrane compared to PDGFRα, as observed under non-permeabilized conditions. This trend was even more evident when comparing biosensors, as PDGFRα-GFP exhibited a higher TIRF/EPI fluorescence signal ratio than PDGFRβ-GFP (Fig. S2c and d).

**Fig. 8:**
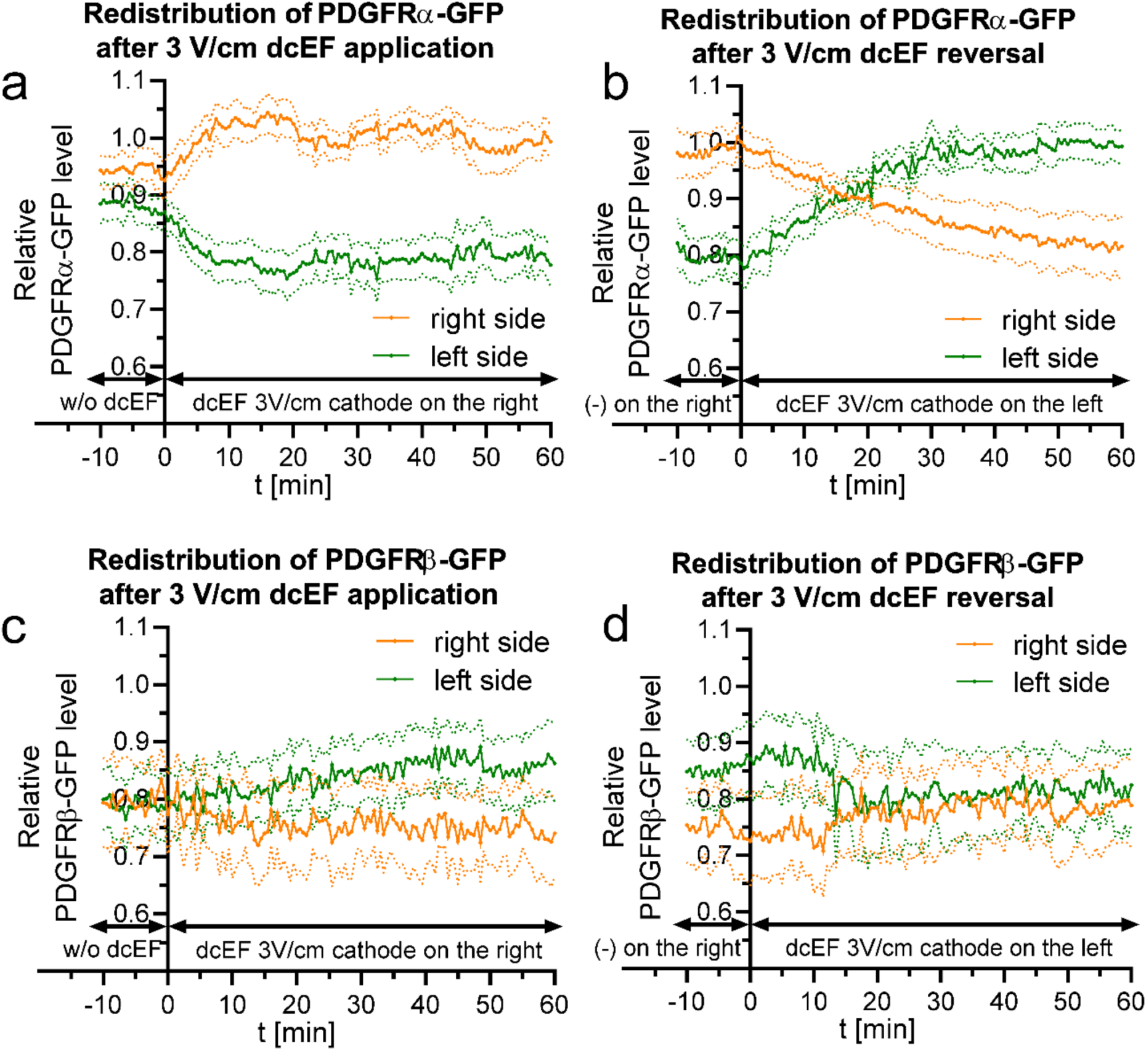
Quantitative analysis of PDGF receptors redistribution following 3 V/cm dcEF application and reversal (**a**, **b**) – PDGFRα; (**c**, **d**)– PDGFRβ; (**a**, **c**) - redistribution of the fluorescent proteins after the application of 3 V/cm dcEF at the 0-minute time point, with the cathode placed on the right side of the field of view. Fluorescence intensities were measured every 30 s for both the right and left sides of the cell (designated at each time point). The values were subsequently normalized by dividing them with the average fluorescence intensity of the entire cell, and are presented as orange and green lines, respectively. (**b**, **d**) - redistribution of the fluorescent protein following the reversal of the dcEF (3 V/cm) polarity at the 0-minute time point, achieved by replacing the electrodes and transferring the cathode to the left side of the field of view. The graphs were constructed as previously (**a**, **c**). The culture medium pH was consistently maintained at 7. The graphs represent average values (± SEM) for *n* = 10 cells (PDGFRα) and *n* = 9 cells (PDGFRβ), each group derived from 3 independent experiments

By comparing the behavior of these receptors under dcEF conditions, we aim to confirm that the effects we observe are specific to membrane proteins and are driven by the electric field. According to theory, cells are present in highly conductive culture medium, while plasma membrane is characterized with substantially higher resistance, which makes intracellular proteins electrically shielded against dcEFs [6]. Obtained results are depicted in the form of images and line profiles (Fig. S3 and S4), and quantitatively (Fig. 8).

The observed changes in PDGFRα receptor localization were less pronounced than those of EGFR but still showed accumulation on the cathode side (Fig. 8a). Upon application of the 3 V/cm dcEF, the level on the cathode side increased by approximately 10%, accompanied by a symmetrical decrease on the anode side. The reversal of equilibrium occurred more than 15 min after changing the dcEF polarity (Fig. 8b).

The comparison with PDGFRβ is particularly interesting because this protein preferentially localizes to intracellular compartments rather than the cell membrane. After applying dcEF, PDGFRβ did not redistribute toward the cathode (the SEM values for the right and left sides overlapped) (Fig. 8c and d). Any observed decrease on the leading edge side can be explained by the formation of a lamellipodium, the signal of which appears less intense in epifluorescence microscopy compared to the contracted trailing edge.

### Distribution of TGFβ receptor 1 during electrotaxis of 3T3 fibroblasts

**Fig. 9:**
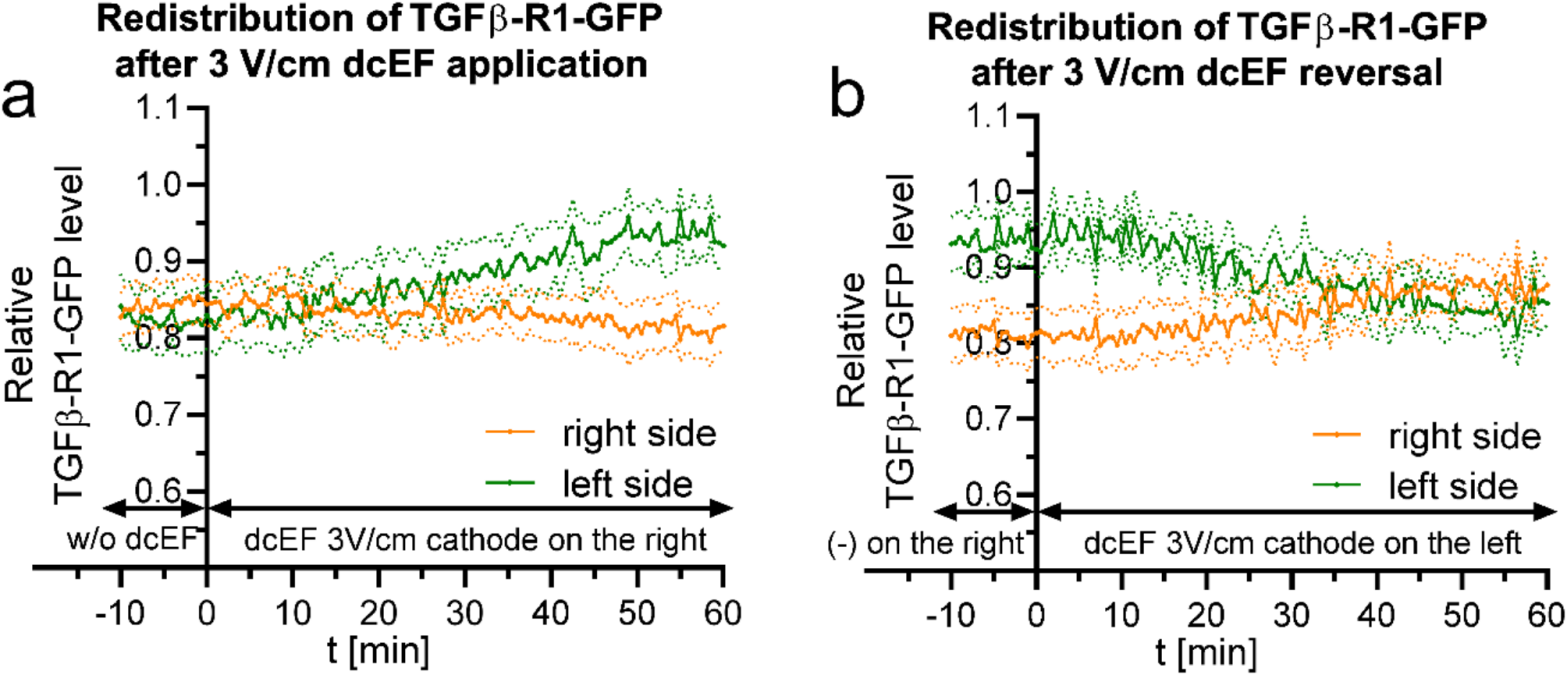
Quantitative analysis of TGFβ receptor 1 distribution following 3 V/cm dcEF application and reversal **(a)** - redistribution of TGFβ R1-GFP after the application of dcEF (3 V/cm) at the 0-minute time point, with the cathode placed on the right side of the field of view. Fluorescence intensities were measured every 30 s for both the right and left sides of the cell (designated at each time point). The values were subsequently normalized by dividing them with the average fluorescence intensity of the entire cell, and are presented as orange and green lines, respectively. **(b)** - redistribution of the fluorescent protein after the reversal of the dcEF (3 V/cm) polarity at the 0-minute time point, achieved by replacing the electrodes and transferring the cathode to the left side of the field of view. The graph was constructed as previously **(a)**. The graphs **(a**,** b)** represent average values (± SEM) for *n* = 8 cells from 3 independent experiments

In our previous work [34], we demonstrated the lack of involvement of TGFβ signaling and its receptor in the electrotaxis of 3T3 fibroblasts. We also visually showed, using images and line profiles, that there was no TGFβ-R1 accumulation on the leading edge of the cells, i.e., the side facing the cathode. In this study, we present a more detailed visual presentation of the TGFβ type 1 receptor distribution in the membrane (Fig. S5) and a quantitative analysis of the process, similar to the analysis performed for the receptors examined above, both in response to the application and reversal of dcEF polarity (Fig. 9).

The obtained results might be somewhat surprising, as they could suggest the accumulation of the receptor on the anode side (Fig. 9a and b). However, it is important to note that the changes on both sides of the cell are not symmetrical, which probably indicates signal variations due to morphological changes and the protrusion of the lamellipodium towards the cathode. Indeed, this distribution resembles that of PDGFRβ, though it is more uniform. Nonetheless, even if there is some redistribution of the receptor in the membrane, it does not correlate with the direction of cell migration. This finding is consistent with our previous results, which indicate that TGFβ signaling is not involved in the electrotaxis of 3T3 fibroblasts [34].

### Effect of silencing specific tyrosine kinase receptors with siRNA on the electrotaxis of 3T3 fibroblasts

**Fig. 10:**
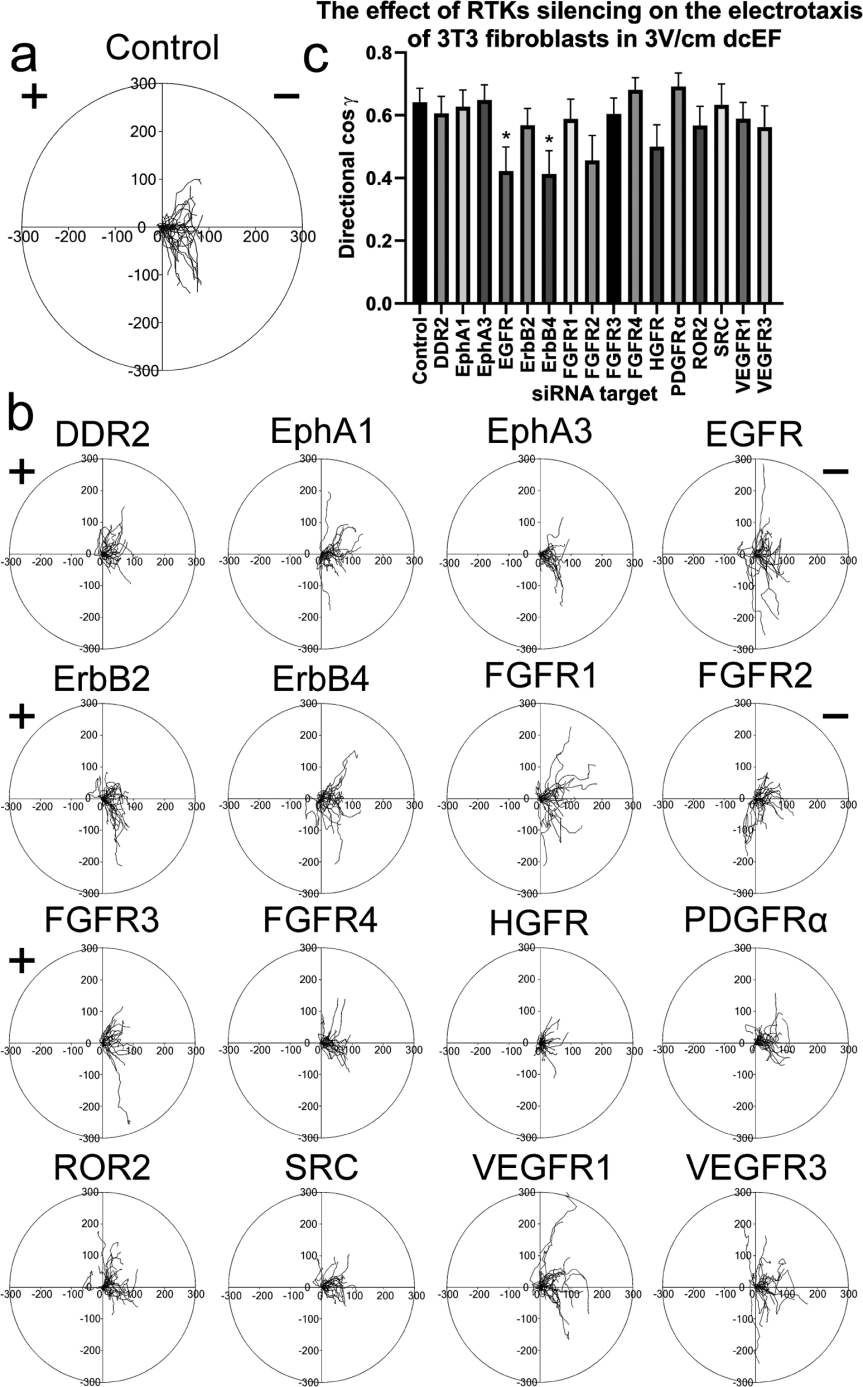
The effect of silencing specific RTK genes (with targeted siRNA) on the electrotaxis of 3T3 fibroblasts. **(a)** Circular diagrams showing composite trajectories of individual cell migration under a dcEF of 3 V/cm. The initial point of each trajectory (constructed from the subsequent 48 cell centroid positions, recorded at 5-minute intervals) was set at the beginning of the coordinate system. The cathode of the dcEF is located on the right side of the diagram. The scale is in µm. **(b)** Circular diagrams, constructed as previously described, showing cells’ electrotaxis 48 h after silencing of specified RTKs with targeted siRNA. **(c)** directionality of cell migration (presented as mean directional cos γ), calculated as the mean (± SEM) for *n* = 30 cells per condition. Control cells were treated with non-targeting siRNA. *Statistically significant differences relative to control (*p* < 0.05)

To assess the role of previously analyzed and other selected membrane receptors with tyrosine kinase activity in the electrotactic response of 3T3 fibroblasts, we used a library of appropriate siRNA constructs to silence their genes. Constructs were selected from the library for genes encoding plasma membrane-localized proteins with receptor activity that are associated with cell migration. Below, we present circular diagrams illustrating the migration of the studied cells and quantitative parameters defining the directionality of their movement in a 3 V/cm dcEF (Fig. 10).

The obtained data suggest that the role of membrane receptors/tyrosine kinases may not be a general rule but is specific to selected receptors, such as the EGF receptor family (i.e., EGFR and ErbB4). Silencing the expression of these genes resulted in a decrease in directional cosine γ by approximately 35%. In contrast, the reduction in the expression of the other genes studied, including PDGFRα, had no effect. This raises the possibility that a pronounced asymmetry in receptor distribution is necessary, as seen with EGFR, whereas the moderate asymmetry observed with PDGFRα may be insufficient.

### Ligand dependence of EGFR signaling in 3T3 fibroblast electrotaxis

**Fig. 11:**
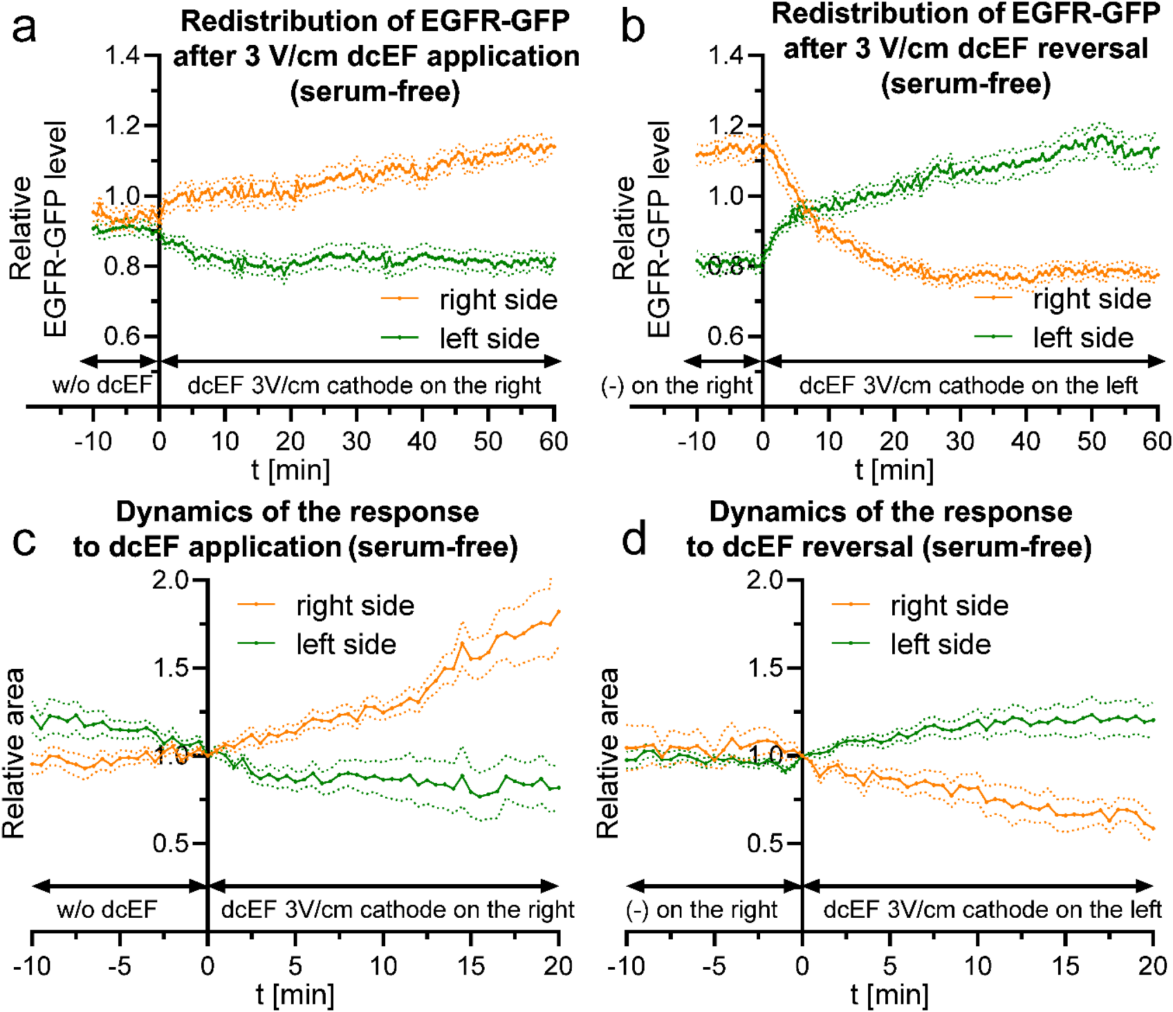
Redistribution of EGFR-GFP and dynamics of 3T3 fibroblasts electrotaxis following dcEF application and reversal in serum-free conditions (**a**) - redistribution of the fluorescent protein after the application of dcEF (3 V/cm) at the 0-minute time point, with the cathode placed on the right side of the field of view. Fluorescence intensities were measured every 30 s for both the right and left sides of the cell (designated at each time point). The values were subsequently normalized by dividing them with the average fluorescence intensity of the entire cell, and are presented as orange and green lines, respectively. (**b**) - redistribution of the fluorescent protein following the reversal of the dcEF (3 V/cm) polarity at the 0-minute time point, achieved by replacing the electrodes and transferring the cathode to the left side of the field of view. The graph was constructed as previously (**a**). The graphs (**a**, **b**) represent average values (± SEM) for *n* = 15 cells from 3 independent experiments; (**c**) - variations in the areas of the cell regions after the application of a dcEF (3 V/cm) at the 0-minute time point, with the cathode placed on the right side of the field of view. The areas of fluorescent cell regions were measured every 30 s for both the right and left sides of the cell (designated at time point 0 min). These values were subsequently normalized to 1 at the time of sectioning and are presented as orange and green lines, respectively. (**d**) - variations in cell region areas after reversing the dcEF polarity at the 0-minute time point, achieved by replacing the electrodes and transferring the cathode to the left side of the field of view. The graph was constructed using the same methodology as in (**c**). The graphs (**c**, **d**) represent average values (± SEM) for *n* = 8 cells from 3 independent experiments. Throughout these experiments, the culture medium pH was maintained consistently at 7

**Fig. 12:**
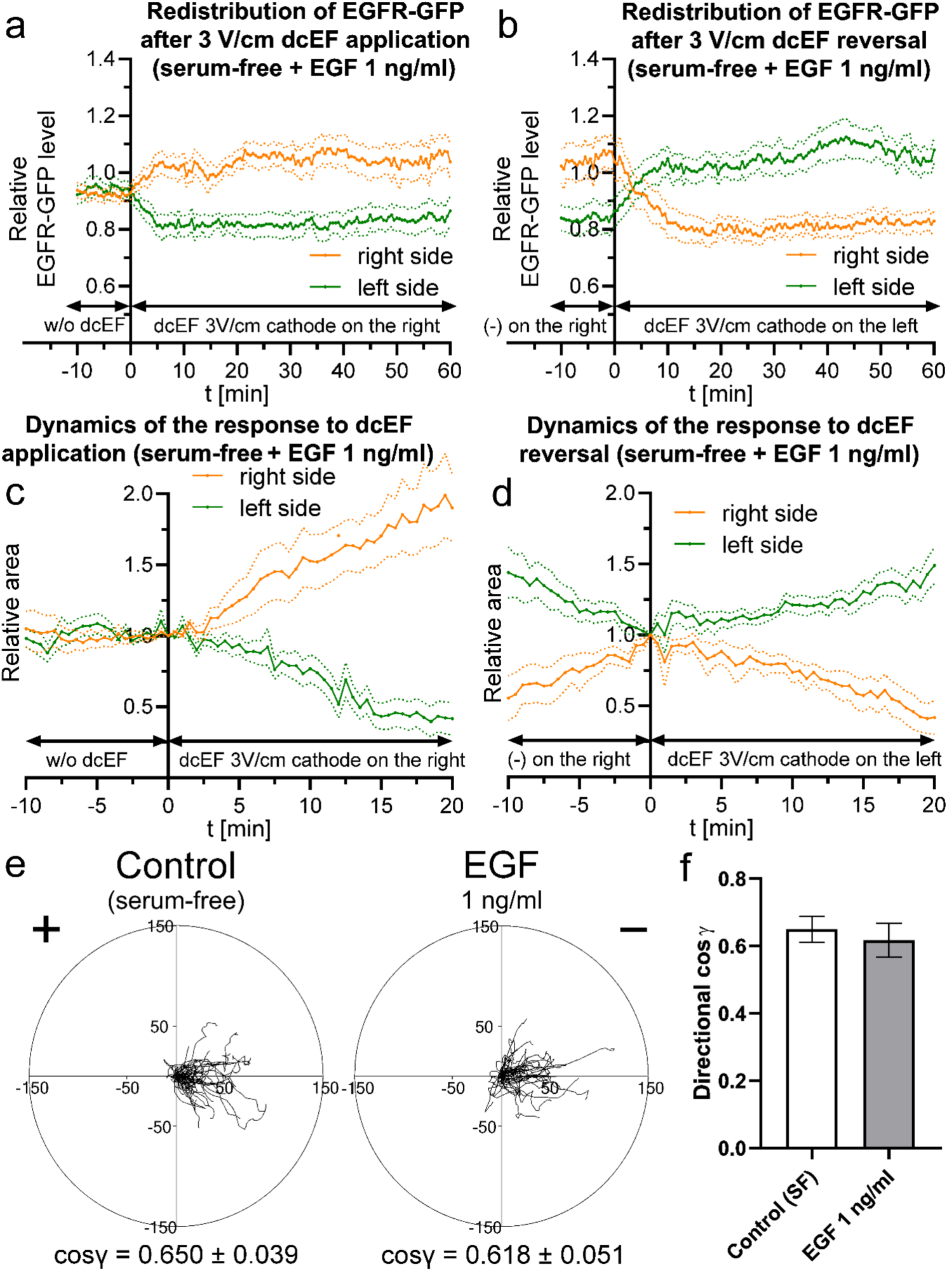
The influence of EGF on the redistribution of EGFR-GFP, long term electrotaxis of 3T3 fibroblasts and its dynamics following dcEF application and reversal (**a**) - redistribution of the EGFR-GFP in the presence of EGF (1 ng/ml) after the application of dcEF (3 V/cm) at the 0-minute time point, with the cathode placed on the right side of the field of view. (**b**) - redistribution of the EGFR-GFP in the presence of EGF (1 ng/ml) following the reversal of the dcEF (3 V/cm) polarity at the 0-minute time point. The graphs (**a**, **b**) were constructed as previously (Fig. 11), and represent average values (± SEM) for *n* = 15 cells from 3 independent experiments; (**c**) - variations in the areas of the cell regions after the application of a dcEF (3 V/cm) at the 0-minute time point, with the cathode placed on the right side of the field of view. (**d**) - variations in cell region areas after reversing the dcEF polarity at the 0-minute time point, achieved by replacing the. The graphs (**c**, **d**) were constructed using the same methodology as in Fig. 11, and represent average values (± SEM) for *n* = 6 cells from 3 independent experiments. Throughout these experiments, the culture medium pH was maintained consistently at 7. (**e**) - Circular diagrams showing composite trajectories of individual cell migration under a dcEF of 3 V/cm in serum-free conditions, or additionally treated with EGF (1 ng/ml). The initial point of each trajectory (constructed from the subsequent 36 cell centroid positions, recorded at 5-minute intervals) was set at the beginning of the coordinate system. The cathode of the dcEF is located on the right side of both diagrams. The scale is in µm. (**f**) - directionality of cell migration (presented as mean directional cos γ), calculated as the mean (± SEM) for *n* = 50 cells per condition

Since our previous experiments involving membrane receptor silencing confirmed the importance of the EGF receptor, we investigated whether this effect is ligand-dependent. We conducted a series of experiments using both serum-free medium and medium supplemented with EGF. We examined whether the presence of an EGFR ligand affects receptor redistribution, the long-term electrotaxis of 3T3 fibroblasts, and the dynamics of this process (Figs. 11 and 12).

In serum-free medium, EGFR-GFP redistribution occurs immediately after the application of dcEF; however, it leads to a lower level of asymmetry compared to conditions with FBS (Fig. 11a). This is accompanied by a highly dynamic response to the applied dcEF, characterized by a rapid increase in cell surface area on the cathode-facing side (Fig. 11c). Upon field reversal (Fig. 11b), equilibrium shifts quickly, with an excess of EGFR-GFP on the ‘new cathode’ side already between the 6th and 7th minute. However, the dynamics of electrotaxis, visualized as changes in surface area relative to the electrodes (Fig. 11d), remain higher than the dynamics of receptor redistribution, similar to what was observed in the presence of serum.

Ligand binding to the receptor can potentially influence both receptor redistribution and, most importantly, its activity and involvement in the studied process. To assess how the presence of the epidermal growth factor affects the examined parameters in our system, we supplemented the serum-free medium with EGF at a concentration of 1 ng/ml (Fig. 12).

It turned out that the presence of the ligand increased the dynamics of redistribution both in response to dcEF application (Fig. 12a) and its reversal (Fig. 12b), without affecting the final level of EGFR-GFP asymmetry. This was accompanied by a noticeably greater electrotactic response dynamics (Fig. 12c and d).

In light of these observations, we examined whether the presence of EGF influences the overall electrotaxis efficiency of the studied cells throughout the experiment. However, neither trajectory-based directionality nor quantitative parameters showed any significant changes (Fig. 12e and f). Similar results were obtained when using a higher concentration of EGF (5 ng/ml), except for an increase in the overall motility of the studied cells from 0.334 ± 0.024 μm/min to 0.454 ± 0.034 μm/min (Fig. S6).

Taken together, these results suggest that the presence of the ligand is not crucial for the role of EGFR in the electrotaxis of 3T3 fibroblasts. Instead, the increased dynamics of the initial response may stem from the ligand’s effect on the speed of receptor redistribution.

### The importance of specific tyrosine kinase receptors in the early phase of electrotaxis of 3T3 fibroblasts

**Fig. 13:**
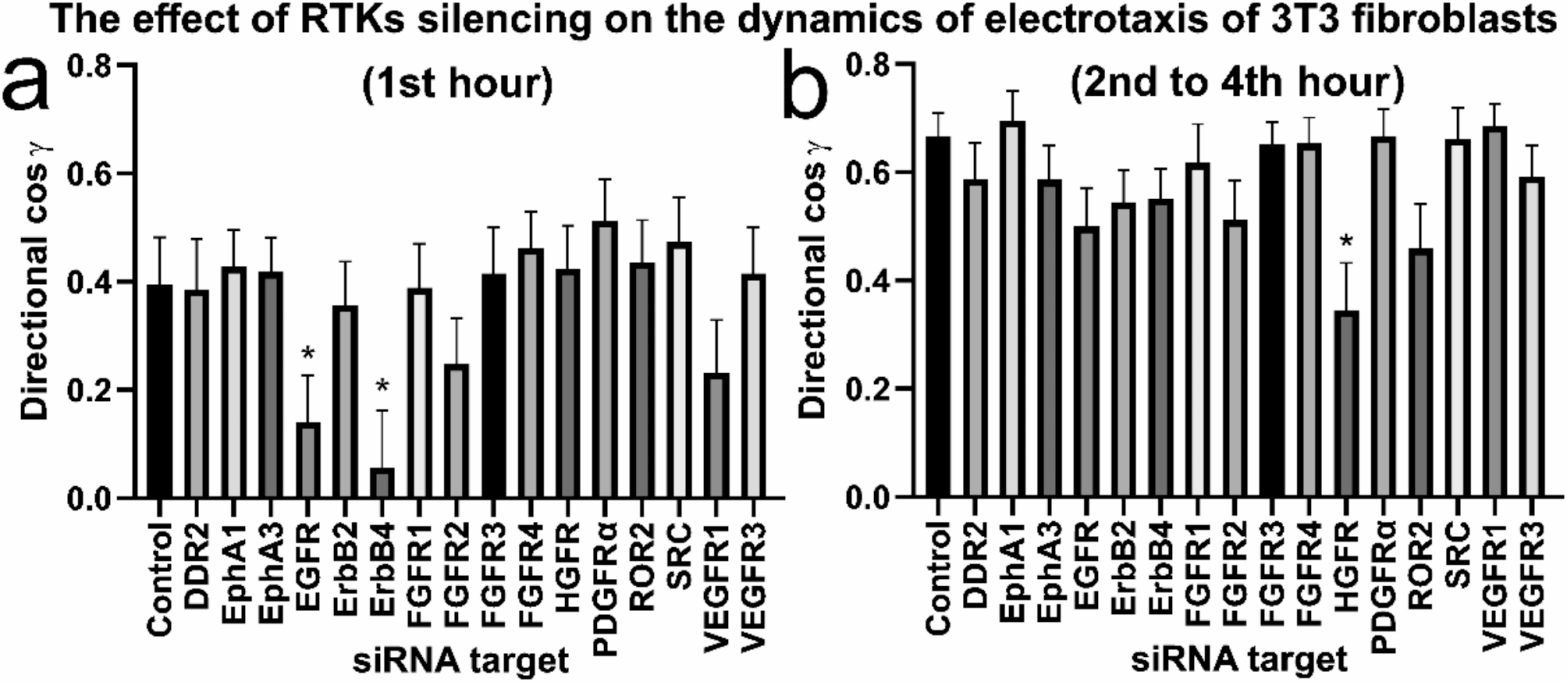
The effect of silencing specific RTK genes (with targeted siRNA) on the dynamics of electrotaxis of 3T3 fibroblasts. Directionality of cell migration (presented as mean directional cos γ), calculated as the mean (± SEM) for *n* = 30 cells per condition, split to (**a**) the initial (1st hour) and (**b**) remaining (2nd to 4th hour) stage of the experiment. Control cells were treated with non-targeting siRNA. *Statistically significant differences relative to control (*p* < 0.05)

In the context of the proposed biphasic mechanism, where membrane receptors are expected to play a role in the later stages of the electrotactic response, analyzing the dynamics of electrotaxis following their reduced expression may be crucial. Additional rationales for this analysis were the surprisingly high dynamics of EGFR redistribution and the increased dynamics of the early electrotactic response in the presence of EGF. The breakdown of results into the first hour of the experiment and the remaining duration provides new insights into the proposed mechanism (Fig. 13).

It was found that the decrease in directionality observed upon EGFR and ErbB4 knockdown originates from disrupted directionality within the first hour of dcEF application (Fig. 13a), while over a longer time scale, directionality is gradually restored. Among the analyzed receptors, only siRNA targeting HGFR resulted in a partial, although statistically significant, decrease in directionality between 2nd and 4th our (Fig. 13b).

In light of these findings, it should be considered that members of the EGF receptor family may also play a role in the early phase of the response to the electric field, which is in line with highly dynamic EGFR-GFR redistribution described here. However, over time, other mechanisms (for example other receptors) may compensate for their absence, ultimately restoring directional migration.

### Interplay between Kir channels and EGFR in electrotaxis regulation

Given the high dynamics of EGFR redistribution, which was even more pronounced under serum-free conditions, and the fact that the knockdown of EGF receptor family genes affected electrotaxis at its early stage, one might question whether EGFR redistribution alone could fully explain such a rapid response of 3T3 fibroblasts to dcEF.

**Fig. 14:**
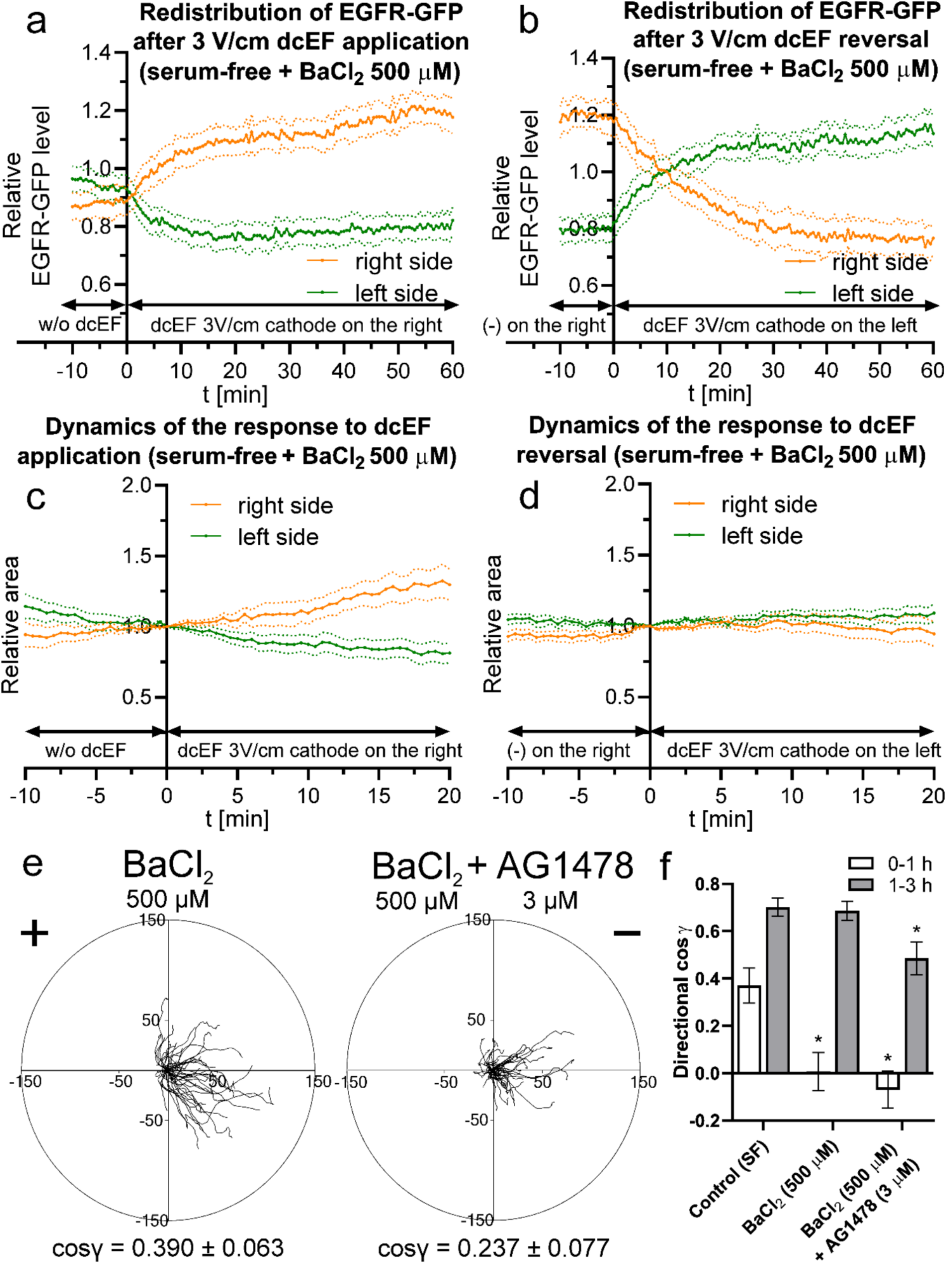
The influence of BaCl_2_ on the redistribution of EGFR-GFP, long term electrotaxis of 3T3 fibroblasts and its dynamics following dcEF application and reversal (**a**) - redistribution of the EGFR-GFP in the presence of BaCl_2_ (500 µM) after the application of dcEF (3 V/cm) at the 0-minute time point, with the cathode placed on the right side of the field of view. (**b**) - redistribution of the EGFR-GFP in the presence of BaCl_2_ (500 µM) following the reversal of the dcEF (3 V/cm) polarity at the 0-minute time point. The graphs (**a**, **b**) were constructed as previously (Fig. 11), and represent average values (± SEM) for *n* = 15 cells from 3 independent experiments; (**c**) - variations in the areas of the cell regions after the application of a dcEF (3 V/cm) at the 0-minute time point, with the cathode placed on the right side of the field of view. (**d**) - variations in cell region areas after reversing the dcEF polarity at the 0-minute time point. The graphs (**c**, **d**) were constructed using the same methodology as in Fig. 11, and represent average values (± SEM) for *n* = 6 cells from 3 independent experiments. Throughout these experiments, the culture medium pH was maintained consistently at 7. (**e**) - Circular diagrams showing composite trajectories of individual cell migration under a dcEF of 3 V/cm in serum-free conditions in the presence of BaCl_2_ (500 µM), or additionally treated with EGF inhibitor– AG1478 (3 µM). The initial point of each trajectory (constructed from the subsequent 36 cell centroid positions, recorded at 5-minute intervals) was set at the beginning of the coordinate system. The cathode of the dcEF is located on the right side of both diagrams. The scale is in µm. (**f**) - directionality of cell migration (presented as mean directional cos γ), split to the initial (1st hour) and remaining (2nd to 3rd hour) stage of the experiment, calculated as the mean (± SEM) for *n* = 50 cells per condition. Serum-free control data originated from Fig. 12.*Statistically significant differences relative to control (*p* < 0.05)

To address this, we designed a set of experiments in which we inhibited Kir channel activity under serum-free conditions, as these channels were previously proposed as key drivers of dynamic electrotaxis in our earlier work [29]. We examined both the effect of BaCl_2_ (500 µM) on EGFR redistribution and its impact on the electrotactic dynamics of 3T3 fibroblasts. (Fig. 14)

As observed, the addition of BaCl_2_ did not inhibit EGFR-GFP redistribution, which was dynamic both upon dcEF application (Fig. 14a) and after field reversal (Fig. 14b). On the other hand, a clear reduction in electrotaxis dynamics was noted (Fig. 14c and d), particularly in response to dcEF reversal. Within the analyzed time window, cell repolarization toward the new cathode was almost entirely absent (Fig. 14d), confirming that both EGFR redistribution and Kir signaling are completely distinct and independent mechanisms in electrotaxis of 3T3 fibroblasts, with Kir channels responsible for the very fast, rapid response to dcEF.

The effect on directionality was also evident over a longer time scale (Fig. 14e), where the reduction in directionality relative to control conditions was pronounced. However, this reduction resulted solely from an effect occurring at the early stage of dcEF application (Fig. 14f).

In addition to BaCl_2_, we further applied an EGFR inhibitor (AG1478), which collectively led to an initial drop in directionality that was not fully recovered throughout the entire experiment (Fig. 14e and f). However, a gradual increase in directionality over time was observed, reinforcing the notion that beyond the contributions of Kir signaling and EGF receptor, additional mechanisms progressively take over, ultimately restoring the electrotactic response.

## Discussion

In our previous research, we proposed a biphasic mechanism of cathodal electrotaxis in 3T3 fibroblasts [29]. It was suggested that their initial rapid response was based on the so-called ionic mechanism, whereas mechanisms involving the redistribution of membrane components would come into play during prolonged exposure to dcEF. We discovered that cells with inhibited inward-rectifying potassium channels activity migrated randomly but manifested recovery of directional movement after 1–2 h of 3 V/cm dcEF application. We hypothesize that the second phase of electrotaxis is based on the redistribution of membrane proteins, such as receptors for chemoattractants. Here, we conducted a series of experiments to determine the extent to which the progression of the process of the redistribution of membrane receptors correlates with the previously observed second phase of the cell response to the electric field.

As we previously confirmed the importance of EGFR signaling in the electrotaxis of 3T3 cells [29], in the current study, we analyzed the redistribution of the EGF receptor linked to GFP in the membrane of 3T3 fibroblasts subjected to dcEF (1–3 V/cm). We demonstrated that this receptor dynamically accumulates on the side of the negative electrode in a manner dependent on the strength of the electric field (Figs. 2 and 3a, and 5a). To date, most of the components studied have also accumulated on this side of the cell, including the ConA receptor [9, 38, 39], the acetylcholine receptor [40], the LDL receptor [11], and the FGF receptor [12]. Importantly, the accumulation of the EGF receptor on the cathode side has also been demonstrated, successively in A431 cells [20], human keratinocytes [19], corneal epithelial cells (CECs) [12, 13], human lung adenocarcinoma A549 cells [41], and neural progenitor cells [42]. It should be noted that all these cells migrated towards the cathode, while MDA-MB-231 cells, which migrate towards the anode, exhibited an opposite polarization of EGFR accumulation [43]. This latter case of receptor accumulation on the anode side is rather rare, although experiments conducted by Allen et al. [6] also indicate a similar localization of an unidentified receptor in the trailing edge of fish keratocytes migrating toward the cathode, which is an interesting finding.

In light of the issue we are exploring in this study, namely the role of the redistribution-based mechanism in response to an electric field and its involvement in the second phase of the biphasic electrotaxis mechanism we previously proposed [29], it is crucial to analyze the dynamics of EGFR receptor redistribution. In the past, conclusions regarding it were drawn on the basis of immunofluorescence staining, which resulted in very different estimates. For example, a higher concentration of EGFR was observed on the cathode-facing side after 30 min in a nonphysiological field strength of 15 V/cm [20]. In corneal epithelial cells (CECs), EGFR accumulated on the cathode side within 10 min of exposure to a dcEF of 3 V/cm [12]. In human keratinocytes, this accumulation occurred as early as 5 min after exposure to electric fields of 1 V/cm [19]. Significant redistribution of EGFR was even reported within the first minute after application of a 5 V/cm dcEF [42]. However, the dynamics of this process were not previously presented in a convincing manner with these methods.

Sarkar et al. [8] thoroughly examined the dynamics of membrane component redistribution, comparing a theoretical model of this process with imaging of tdTomato-GPI redistribution, a small protein anchored in the cell membrane. This protein moved toward the cathode, reaching saturation within 7 min of dcEF application, reflecting the calculations of the theoretical model for rounded CHO cells with a diameter of 15 μm. In our case, the signal stabilized after approximately 30 min in a dcEF of 3 V/cm, but at a much higher level of asymmetry (Fig. 3a). According to the model developed by Sarkar et al. [8], this trend is understandable, as our system studies a much larger protein with a transmembrane α-helix, and we used fully spread 3T3 fibroblasts with a diameter exceeding 50 μm. The level of asymmetry is difficult to compare with other studies on EGFR redistribution, as the semi-quantitative measurements used in those studies were performed with different methodologies. However, the asymmetry reached a 3-fold advantage on the cathode side in a nonphysiological dcEF of 15 V/cm [20], while in our case, we observed over a 2-fold higher level of EGFR-GFP on the cathode side in a dcEF of 3 V/cm (Fig. 3a) and approximately a 1.5-fold increase in a dcEF of 1 V/cm (Fig. 5a). We previously showed that when the ionic mechanism of electrotaxis was disrupted, directional migration was restored after more than an hour of exposure to a 3 V/cm dcEF, whereas it did not occur at 1 V/cm [29]. Our current observations, interpreted in the context of the biphasic mechanism of electrotaxis that we proposed there, suggest that a sufficiently high asymmetry in receptor redistribution is necessary to elicit directional cell migration, requiring both a strong enough dcEF and sufficient time. However, even in this situation, the observed dynamics do not fully align with the secondary phase of the proposed biphasic mechanism. On the other hand, it is also hard to reconcile with the previous theory that even the smallest inequality of membrane receptor redistribution is able to drive 3T3 fibroblasts electrotaxis, in which the authors attempted to explain the electrotaxis phenomenon entirely with this mechanism [14]. Such a hypothesis cannot explain our observations discussed below.

Due to the limitations of previously used experimental setups, no one has yet investigated the dynamics of membrane protein redistribution occurring after the reversal of the electric field, which may, however, be crucial for further interpretation of the role of the studied mechanism. In our work, we demonstrated that after the electrodes are switched and the cathode is positioned where the anode previously was, there is a dynamic flow of protein to the opposite side of the cell, which begins immediately after the event (Figs. 2c and 3b). This process can be particularly dynamic because, at this point, both diffusion and the electric field favor the outflow of the protein from the region where it was previously accumulated. Nevertheless, in light of the hypothetical mechanism based on the redistribution of membrane receptors, it is crucial to determine when the receptor begins to predominate on the ‘new cathode’ side, thus determining the formation of the cell’s leading edge [10]. We demonstrated that in our system this occurs 13 min after the reversal of the dcEF at 3 V/cm (Fig. 3b).

These results need to be confronted with the analysis of electrotaxis dynamics. Our analysis based on changes in cell surface area on both sides indicates an almost immediate shift in lamellipodium formation to the ‘new cathode’ side (Figs. 3d and 4b), as we have previously shown in other studies, including for the cells examined here [17, 29]. This supports the hypothesis that another, faster detection mechanism must be responsible for the first, very rapid response to the electric stimulus. Our previous research suggests the involvement of specific ion channels in this process [29], but we cannot rule out a rapid detection mechanism based on the electromechanical phenomenon proposed by Hart et al. [44]. They pointed to the more credible role of this mechanism compared to the electrodiffusion studied here, as the application of a low-intensity alternating current electric field (acEF) disrupted the influence of a much stronger dcEF on the electrotaxis of fast-migrating fish keratocytes. Nevertheless, the significance of protein redistribution-based mechanisms is supported by experiments by Zajdel et al. [45], showing the averaging of electric fields alternately applied perpendicularly to each other, leading to the collective migration of MDCK cells at a 45-degree angle, as well as studies demonstrating the effectiveness of low-frequency pulsed electric fields in inducing keratinocyte electrotaxis and the occurrence of movement direction memory after the cessation of such a stimulus [46]. Therefore, it seems that at least in slow-migrating cells, such as the 3T3 fibroblasts studied here, the mechanism based on membrane protein redistribution has strong potential to account for the prolonged response to the electric field and the stabilization of electrotaxis during physiological processes, rather than the first rapid response to applied or reversed dcEF.

Since we demonstrated and thoroughly characterized the process of EGFR-GFP redistribution and acknowledged its role in determining a stable electrotactic response, we then focused on the mechanism underlying this redistribution. According to theory and established mathematical models, the driving forces behind the electromigration of proteins in the cell membrane are electrophoresis and electroosmosis [8–10]. Both of these processes should respond differently to changes in acidity, which affect the charge of the proteins under study and the net charge associated with the cell surface [6, 8]. In the course of our research, we observed a decrease in redistribution dynamics when the pH was lowered to 6 (Fig. 6a) and an opposite effect when the pH was raised to 8 (Fig. 6b). The acidification of the medium caused the signal to stabilize longer - even for the entire duration of the dcEF application, which lasted 60 min (Fig. 6a), whereas at pH 8, stabilization occurred as early as 20 min after the application of dcEF (Fig. 6b). Notably, the time required to reverse the equilibrium to the ‘new cathode’ side was reduced to 6 min after the reversal of dcEF (Fig. 6d). All of this indicates an increase in redistribution dynamics with the alkalization of the environment.

The result was somewhat surprising, as an opposite effect on EGFR-GFP accumulation was expected due to acidification of the medium, similar to what was observed in the studies by Sarkar et al. [8], where it enhanced tdTomato-GPI accumulation in both experimental and theoretical models, with electrophoresis favoring electromigration toward the cathode. The differences can likely be attributed to the nature of the protein used in the studies. Although the basic isoelectric point (pI) of EGFR is calculated to be 6.26, it can be lower as more glycosylation sites, especially those containing sialic acid, are occupied [8]. Furthermore, binding of the receptor to its ligand, EGF, which has a pI of 4.60, may be crucial [47]. Such a complex, even at an environmental pH of 6, would have a net negative charge, resulting in the protein having a negative charge across the entire pH range of 6–8, leading to electrophoresis towards the positive electrode, that is the anode, which was not observed experimentally. Regardless of the environmental acidity, EGFR-GFP accumulates on the cathode side, with a particularly pronounced effect at pH 8 (Figs. 3 and 6). This alkalization would favor the reduction of the cell’s zeta potential, thereby enhancing electroosmosis, which here is definitely a key to redistribution. The zeta potential at pH 7 is negative for most tissue cells, and most of the groups responsible for this have a pI lower than 6 [8]. Consequently, it remains negative throughout the tested range, with a particularly strong effect at pH 8.

Different observations were made by Allen et al. [6], where acidification of the environment resulted in the inhibition of electrotaxis in fish keratocytes. This, combined with the positive zeta potential of these cells, was interpreted as the critical role of a hypothetical membrane component with an isoelectric point of around 6, which, after acidification, ceased to undergo electrophoresis towards the anode. Likewise, the electrophoresis of heparan sulfate to the anode was also suggested to be key for the electrotaxis of fetal neural progenitor cells (fNPCs), fNPC-derived astrocytes, and brain tumor-initiating cells (BTICs), regardless of their direction of movement [48].

On the other hand significance of electroosmosis as a key driving force for the redistribution of membrane components is highlighted by experiments employing different methodological approaches. For example, the reversal of ConA redistribution through preincubation with neuraminidase, which cleaves sialic acid residues, or by saturating the membrane with DiI, both of which result in the reversal of the net charge of the cell membrane [9]. Additionally, the reversal of the migration direction of fish keratocytes by small viscous polymers in the cell environment is explained by the disruption of the baseline electroosmotic flow and the promotion of opposing electrophoresis [49].

Electroosmosis also occurs near the substrate on which the cell is situated [6]. The surface of glass has a net negative charge, leading to the accumulation of positive counterions that are actively drawn to the cathode, along with their hydration shells, causing the generation of laminar flow in that direction [6]. This may explain the particular dynamics of EGFR-GFP redistribution in the basal membrane of cells, as observed using TIRF microscopy (Fig. 7a and c). At the same time, coating the substrate with poly-L-lysine, which imparts a positive charge, reduced the dynamics of redistribution, although it still occurred toward the cathode (Fig. 7b and d). It seems that the laminar flow on the surface of the cell membrane, which is crucial for the segregation of proteins on its surface, plays a key role here.

In the past, it has been shown that coating the substrate with poly-L-lysine reversed the electrotropism of *Xenopus* neurites from cathodal to anodal [50]. Similarly, BTICs on a poly-L-ornithine/laminin coated surface migrated in the opposite direction (to the anode) compared to when they were embedded in a matrix of hyaluronic acid and collagen, indicating the crucial importance of the charge of the substrate on which they are located [51]. Interestingly, a similar substrate modification did not alter the electrotaxis of fish keratocytes in the studies mentioned above, despite the proven effect on reversing the electroosmotic flow on poly-L-lysine coated surfaces [6].

Our subsequent experiments confirmed that the observed redistribution of receptors relates only to proteins localized in the cell membrane. Among the PDGFRα and PDGFRβ pair, only the former accumulated on the cathode side (Fig. 8 and Fig. S3), although with less intensity than EGFR, achieving an excess of approximately 1.25 compared to the anode-facing side (Fig. 8a). The lack of asymmetry in PDGFRβ was expected, as in our cells, it was primarily located in the membranes of intracellular compartments, rather that in plasma membrane (Fig. S2 and S4). The literature confirms that the cell membrane possesses insulating properties, with its resistance estimated to be in the tens of kiloohms per cm². Combined with the small size of cells and the existing electrical polarization of the cell membrane, this means that externally applied dcEF only slightly affects components located in the cytoplasm [6, 52].

Using the developed research model, imaging and quantitative analysis, we also thoroughly examined the changes in the membrane distribution of the TGFβ type I receptor (TGFβ-R1). This receptor was of particular interest to us because, in a previous publication, we demonstrated its lack of involvement in the electrotaxis of 3T3 fibroblasts, as well as the absence of significant redistribution in the cell membrane at selected time points [34]. On the other hand, we have previously shown an importance of TGFβ-R1 signaling for anodal electrotaxis of Human Bronchial Fibroblasts [53], as well as earlier literature had shown an involvement of the TGFβ type II receptor in the electrotaxis of CEC cells together with its slight redistribution in their membrane toward the cathode [12]. In our case, we did not observe accumulation of TGFβ-R1-GFP on the cathode side (Fig. S5), and delicate changes in signal between the right and left sides were not fully symmetric (Fig. 9), which we suspect may be due to morphological changes of cells rapidly responding to the applied dcEF. Thus, it seems that the occurrence of redistribution in the cell membrane is not a universal feature for all receptors localized in the cell membrane. This is supported by the in-depth considerations in the work of Sarkar et al. [8], where the specific characteristics of proteins that favor efficient electromigration of membrane proteins are discussed in detail. They noted the relatively small extracellular domain of TGFβ-R1, which does not favor efficient electroosmosis [8].

The final analysis of the impact of inhibiting the expression of selected genes for membrane receptors with specific siRNA allows us to compare the significance of their presence in the electrotaxis of 3T3 fibroblasts with previous observations. Among the inhibited genes, an effect on electrotaxis was observed for two representatives of EGF receptor family, including EGFR, whose redistribution to the cathode was thoroughly characterized, as well as ErbB4 (Fig. 10). In both cases, the decrease in directionality was statistically significant but was not complete, indicating that none of these components alone is responsible for the electrotaxis of the cells studied (Fig. 10c). The importance of EGF receptors has previously been demonstrated using pharmacological methods, where inhibition of their kinase activity significantly reduced movement directionality in dcEF [19, 29, 41].

Given the documented role of PDGFRα in chemotaxis, such as in lung fibroblasts [54], as well as its previously shown redistribution to the cathode (Fig. 8a and Fig. S3), we could have suspected its role in the electrotaxis of the cells studied here. However, siRNA for gene of this receptor did not reduce movement directionality (Fig. 10b and c). It may appear that the level of asymmetry observed for this protein is not sufficient to independently drive the directional movement of 3T3 fibroblasts in an electric field. The lack of a significant decrease in directionality may also be surprising for the studied FGF receptor types (Fig. 10b and c), given the previously demonstrated importance of this growth factor and the redistribution of its receptor to the cathode in the electrotaxis of CEC cells [12].

Since our experiments confirmed the involvement of EGFR in electrotaxis, and we characterized its redistribution in detail, we next examined how these processes depend on the presence of its ligand, EGF. Experiments conducted in serum-free medium yielded intriguing observations, as EGFR distribution asymmetry was lower under these conditions (Fig. 11a). Our observations can be compared with findings of Zhao et al. [12], who reported a strong dependence of EGFR asymmetry on serum presence, with no significant asymmetry observed in 1.5–3 V/cm electric fields under serum-free conditions. However, their use of immunostaining or fluorescent ligand at fixed time points may have limited the detection of subtle redistribution events. In our study, while EGFR asymmetry was lower in serum-free conditions, it remained detectable. Interestingly, although the addition of EGF enhanced EGFR redistribution dynamics to some extent, it did not significantly increase the final asymmetry level (Fig. 12a and b). Based on this, the EGFR asymmetry level appears to be more dependent on serum than on EGF itself, which was not verified by Zhao et al. [12]. Such an effect could be related to changes in membrane fluidity and the submembrane cytoskeletal structure, which influence membrane protein mobility [55]. Additionally, significant alterations in medium viscosity could also contribute to this phenomenon [56].

The slight increase in redistribution dynamics following EGF binding mentioned above (Fig. 12a and b) may be attributed to enlargement of the extracellular domain, which plays a crucial role in electroosmosis-driven redistribution [8]. However, given the relatively small size of EGF [57], this effect remains limited. Furthermore, receptor activation and cytoskeletal interactions could be expected to reduce EGFR mobility due to its association with actin-binding proteins and integration into larger signaling complexes, which typically restrict lateral diffusion in the membrane [58]. This anchoring effect stabilizes receptor localization and facilitates signal transduction. However, this was not observed within the tested concentration range (1–5 ng/ml), suggesting that under these conditions, cytoskeletal constraints may not be the primary factor influencing EGFR redistribution.

Although initial electrotaxis dynamics (Fig. 12c) correlated with higher redistribution dynamics under these conditions (Fig. 12a), the presence of EGF did not affect overall migration directionality in dcEF (Fig. 12e and f), suggesting a ligand-independent process. If EGFR operates in a ligand-independent manner in this system, its redistribution and local clustering could lead to receptor activity enhancement and spontaneous activation. A relevant reference in this context is a study indicating that EGFR can be maintained in an inactive state within lipid rafts, with receptor release from these domains facilitating close interactions that may contribute to activation [59]. Additionally, previous research has demonstrated that increased receptor density in cells overexpressing EGFR can lead to enhanced activity [60]. Hypothetically, a similar phenomenon may occur when a significant surplus of EGFR accumulates on one side of the cell due to electric field-driven redistribution.

Some of the obtained results, like the unexpectedly high dynamics of EGFR redistribution, additionally enhanced upon EGF stimulation, could suggest the involvement of a redistribution-based mechanism at an earlier stage than proposed by our biphasic model. A refined perspective on the electrotaxis mechanism emerged from an analysis of the siRNA experiment results divided into two time intervals, which was motivated by the need to distinguish between early and late effects of receptor silencing. This approach reveals that, when members of the EGF receptor family are silenced, the impact on migration directionality is already evident within the first hour of electric field application (Fig. 13a). Moreover, it is surprising that in the second phase of the experiment (Fig. 13b), the inhibition of these receptors no longer influences migration directionality, indicating cellular adaptation and the activation of alternative mechanisms. One potential candidate for this compensatory response is HGFR, as its absence results in a partial yet significant decrease in directionality between the 2nd and 4th hour (Fig. 13b). However, it is unlikely to be the sole factor involved.

Despite EGFR’s involvement from the beginning, as mentioned earlier it alone does not fully account for the exceptionally rapid response to the applied electric field, particularly in the first few minutes of exposure and in response to polarity reversal. Due to highly dynamic reversal of EGFR equilibrium in serum-free medium, we additionally inhibited Kir channels via BaCl_2_ to evaluate their contribution to the immediate response to dcEF application and reversal. Most strikingly, inhibition of Kir channels nearly abolished electrotaxis within the first 20 min after field reversal (Fig. 14d), despite pronounced EGFR redistribution (Fig. 14b). This confirms that both of these mechanisms function independently, with Kir channels playing a particularly crucial role when cell repolarization is necessary. As in our previous paper [29], blocking Kir channels led only to a transient drop in electrotaxis, with directionality gradually returning to control values (Fig. 14e and f). To determine if this could be due to redistributed receptor activity, a combined inhibition of Kir channels and EGFR using AG1478 was tested, revealing that directional migration did not fully recover (Fig. 14e and f). This incomplete restoration compared to the siRNA inhibition (Fig. 13b) may result from the greater efficacy of the pharmacological inhibitor compared to gene silencing, which, when using siRNA, is never fully effective [61]. Additionally, the lack of full recovery in the presence of AG1478 may be attributed to its limited specificity for EGFR, as it also inhibits ErbB4 [62, 63]. Since each siRNA experiment targeted only a single receptor at a time, potential compensatory mechanisms cannot be excluded.

Although the study was conducted with great care and allowed us to answer many questions related to the mechanism of electrotaxis, it has several limitations that should be acknowledged. Most experiments were conducted in serum-containing conditions with uncontrolled growth factor concentrations, potentially influencing receptor mobility through activation-induced dimerization or steric hindrance affecting electroosmosis. Serum-free experiments with EGF confirmed ligand effects on receptor dynamics, though the changes were minor. One limitation is the incomplete efficiency of siRNA-mediated gene silencing, which may have influenced receptor knockdown effects. Future studies on key receptors could use full gene deletion and stable cell lines. Interpretation of receptor knockdown experiments requires caution due to variability in receptor expression among cells. To improve broad relevance, additional studies on diverse cell lines or engineered overexpression models are needed.

Additionally, our study is constrained by some analytical limitations, as our method for studying electrotaxis dynamics only captures a 20-minute window post-application or reversal of dcEF, restricting interpretation with longer-term redistribution effects. As a result, the spatial scale also differs from receptor redistribution studies, complicating direct comparisons. Similarly, TIRF microscopy experiments had to be shortened due to a limited field of view and a risk of cells migrating out of the observable area over prolonged experiments. Implementing a microscope system capable of real-time cell tracking and maintaining full focus would improve data acquisition and interpretation.

## Conclusions

Our detailed analyses of the dynamics of membrane protein redistribution performed for the first time with such specificity for proteins as large as EGFR and PDGFRα indicate that these proteins begin to move immediately after the application of dcEF. However, a relatively long time passes before a clear asymmetry between the poles of the cell is achieved. Although the observations made could fully explain the occurrence of a dynamic response to the applied dcEF, they cannot account for the immediate response to the reversal of dcEF, which clearly precedes the reversal of protein concentration equilibrium.

In verifying the previously proposed biphasic model, we expected the dynamics to fully correlate with the recovery of directional cell movement following Kir inhibition after at least 1 h of dcEF application. However, it turned out that the proteins accumulate more dynamically. Moreover, although the role of EGFR in 3T3 fibroblasts in long-term electrotaxis appeared to be independent of ligand binding, its presence slightly increased electrotaxis dynamics. Furthermore, we discovered that EGFR and ErbB4 contribute to the electrotaxis of 3T3 fibroblasts as early as within the first hour of dcEF exposure, aligning with the relatively dynamic EGFR redistribution. Nevertheless, these receptors cannot account for the initial response to dcEF application and reversal, which was significantly impaired when Kir channels were inhibited, despite redistribution remaining unaffected. Interestingly, even when both proposed components - Kir channels, and EGF receptor family - are inhibited, 3T3 fibroblasts retain the ability to adapt and partially regain electrotactic behavior, suggesting the involvement of additional compensatory mechanisms.

In conclusion, we were able to confirm that dcEF induces a marked asymmetry of selected receptors for chemoattractants through the phenomenon of electroosmosis, determining a mechanism that has all the potential to participate in the early response to dcEF and stabilize long-term electrotaxis. However, electrotaxis appears to be more complex than the previously proposed biphasic model, suggesting a multimodal regulatory mechanism.

## Electronic supplementary material

Below is the link to the electronic supplementary material.

The supplemental materials include figures illustrating the validation of the siRNA knockdown procedure (Fig. S1), a differential analysis of PDGFRα/β localization within cells (Fig. S2), and the redistribution of PDGFRα/β (Fig. S3 and S4) as well as TGFβR1 (Fig. S5). We also show the effect of a higher EGF concentration (5 ng/ml) on EGFR redistribution and electrotaxis (Fig S6). Additionally, supplementary videos provide detailed depictions of EGFR redistribution (Additional file 1) and the dynamics of 3T3 fibroblast electrotaxis (Additional file 2). The latter is further presented with visualizations of actin cytoskeleton dynamics (Additional file 3) and focal adhesion turnover during electrotaxis (Additional file 4).
Supplementary Material 1: Additional file 1 -.mov Visualization of EGFR-GFP redistribution in response to dcEF. 3T3 fibroblasts transfected with a plasmid encoding EGFR-GFP were observed using fluorescence microscopy before and during the application of a dcEF (3 V/cm). Both a monochromatic (green) signal and fluorescence in pseudocolors are presented, with a colored bar indicating the dynamic range. The recording begins with a 10-minute observation of cells under isotropic conditions. Subsequently, a dcEF was applied for 60 min with the cathode positioned on the right side. After this period, the dcEF polarity was reversed by switching the electrodes, placing the cathode on the left side. Images were captured every 30 s. The frame rate is 15 fps, and the scale bar represents 50 μm.

Supplementary Material 2: Additional file 2–.mov - Visualization of the 3T3 fibroblasts electrotaxis dynamics. Cells expressing GFP were recorded using fluorescence microscopy following the application and reversal of a 3 V/cm dcEF. The green image of the cell at the specified time point was overlaid with the red image of the cell at the reference time point, where the dcEF of 3 V/cm was applied (0 min) or reversed (60 min). As a result, the green color denotes a region covered, while the red color denotes a region released by the cell between the reference time and the specified time point. Images were captured every 30 s. The frame rate is 15 fps. The scale bar represents 50 μm.

Supplementary Material 3: Additional file 3–.mov - The dynamics of the actin cytoskeleton during electrotaxis of 3T3 fibroblasts– response to application and reversal of dcEF. Cells expressing Life-Act (indicating F-actin) were recorded every minute for 3 h and 20 min. After the initial 10 min under isotropic conditions, a dcEF of 3 V/cm was applied with the cathode positioned on the right side for 60 min. The polarity was then reversed by moving the cathode to the left side for the next 80 min, after which the cathode was returned to the right side for the remainder of the recording. The green signal was captured with epifluorescence, while the red channel was recorded using TIRF microscopy. The frame rate is 15 fps.

Supplementary Material 4: Additional file 4 -.mov - The dynamics of focal adhesions during electrotaxis of 3T3 fibroblasts - response to dcEF application and reversal. Cells expressing mEmerald-Vinculin (indicating focal adhesions) were recorded every minute for 2 h and 30 min. After an initial 10 min under isotropic conditions, a dcEF of 3 V/cm was applied with the cathode positioned on the right side for 100 min. The polarity was then reversed by moving the cathode to the left side for the next 40 min. The green signal was captured with epifluorescence, while the red channel was recorded using TIRF microscopy. The frame rate is 15 fps and the scale bar represents 25 μm.

Supplementary Material 5

## Data Availability

The datasets used and analyzed during the current study are available from the corresponding author on reasonable request.

## Abbreviations

dcEF: direct current electric field
EGFR: Epidermal growth factor receptor
PDGFRα/β: Platelet-derived growth factor receptor alpha/beta
TEP: Transepithelial potential
TGFβR1: Transforming growth factor beta-receptor 1
TIRFM: Total internal reflection fluorescence microscopy
