# Supplementary material for: The dynamics of chemoattractant receptors redistribution in the electrotaxis of 3T3 fibroblasts
Source: Cell Commun Signal. 2025 Apr 8;23:173. doi: 10.1186/s12964-025-02165-4 (PMC11980103; doi:10.1186/s12964-025-02165-4)
Supplement: Supplementary file 5 — Supplementary Material 5 [file 12964_2025_2165_MOESM5_ESM.docx]

**Supplementary methods**

**Immunofluorescence staining and microscopic imaging of PDGFRs**

3T3 fibroblasts were seeded at 1.0 × 10⁴ cells per well of a 12-well plate containing 15 × 15 mm glass coverslips and cultured in complete culture medium under standard conditions
for 24 hours. Then, cells were fixed with 3.7% formaldehyde for 10 min at room temperature (RT) and prepared under two conditions: non-permeabilized (for membrane-localized proteins) and permeabilized (for total cellular pool, 0.1% Triton X-100 for 5 min). Samples were blocked in 3% BSA/PBS for 90 min and incubated overnight at 4°C with primary antibodies: PDGFRβ (rabbit polyclonal, 1:100, Thermo Fisher PA5-96085) and PDGFRα (rat monoclonal,
1:200, Thermo Fisher 14-1401-82). After washing, Alexa Fluor 488-conjugated secondary antibodies (chicken anti-rabbit and goat anti-rat, both 1:400) were applied for 2 h at RT,
along with Hoechst 33258 (2 µg/mL) (Thermo Fisher Scientific) for nuclear staining. After washing coverslips were mounted in Dako fluorescence mounting medium (Agilent Technologies, Santa Clara, CA, USA) and imaged using a Leica DMI6000B fluorescence microscope, equipped with DFC360FX CCD camera, HCX PL APO 40×/1.25 OIL objective, and EL6000 metal-halide illuminator, working under the control of LAS X 3.7 (all from Leica, Wetzlar, Germany).

Quantitative analysis of obtained images was performed in Fiji ImageJ 1.54f. Cells were manually outlined, and fluorescence intensity was measured for at least 50 cells per condition, together with background signal, which was later subtracted from the cell measurements. The membrane-to-total fluorescence ratio was calculated (i.e., non-permeabilized/permeabilized). Error propagation was applied using the SEM of particular groups.

Additionally, TIRF/EPI fluorescence microscopy imaging was performed as described previously, with a penetration depth of 70 nm for the TIRF channel and a 0° incident angle
of the 488 nm laser for the EPI channel, on cells transfected with plasmids encoding PDGFRα-GFP and PDGFRβ-GFP, and seeded onto CELLview culture dishes with a glass bottom (Greiner Bio-One, Frickenhausen, Germany). Quantitative analysis was conducted
as described above, with fluorescence measured in both channels for each cell, and the TIRF-to-EPI fluorescence ratio was determined to assess relative membrane localization.

**Western-blot analysis**

3T3 fibroblasts were seeded at 6.0 × 10⁴ cells per well of a 12-well plate, and after 24 hours were transfected with 25 nM siRNA targeting mouse Gapd (#D-001830-02-05) or green fluorescent protein targeting control (#P-002048-01-20) using Dharmafect I as the transfection reagent (all reagents from Dharmacon) according to the manufacturer's instructions. After 48 hours, cells were washed with phosphate-buffered saline (PBS; Gibco/Thermo Fisher Scientific) and lysed with RIPA lysis buffer (Sigma-Aldrich) with the addition of protease
and phosphatase inhibitors (Thermo Fisher Scientific). Protein concentration was determined using a BCA assay (Thermo Fisher Scientific) according to the protocol provided by
the vendor. Protein samples (10 μg/line) were loaded onto Miniprotean TGX Gel, 4-15% (Bio-Rad) and separated by electrophoresis. Proteins were then transferred to polyvinylidene fluoride (PVDF) membrane (Bio-Rad) by semi-dry transfer at 25V, 1.3A for 7 minutes in the Trans-Blot Turbo Transfer System (Bio-Rad). Membranes were incubated in 3% BSA in TBST (0.05 (v/v) Tween 20 in Tris-buffered saline) for 1 hour at room temperature (RT) to block nonspecific binding sites. The membranes were then incubated with primary antibodies against GAPDH (mouse monoclonal IgG1, #MA5-15738; Invitrogen/Thermo Fisher Scientific) and β-tubulin (mouse monoclonal IgG2a, #MA5-16308; Thermo Fisher Scientific) overnight at 4°C. The levels of proteins were then detected with a horseradish peroxidase (HRP)-conjugated secondary antibody (horse anti-mouse IgG, #7076; Cell Signaling Technology) after incubation with the membranes for 1 hour at RT. The signal was detected using the chemiluminescent HRP substrate (Merck) in the ChemiDoc XRS + imager (Bio-Rad). Densitometric analysis was performed using Quantity One software (Bio-Rad).


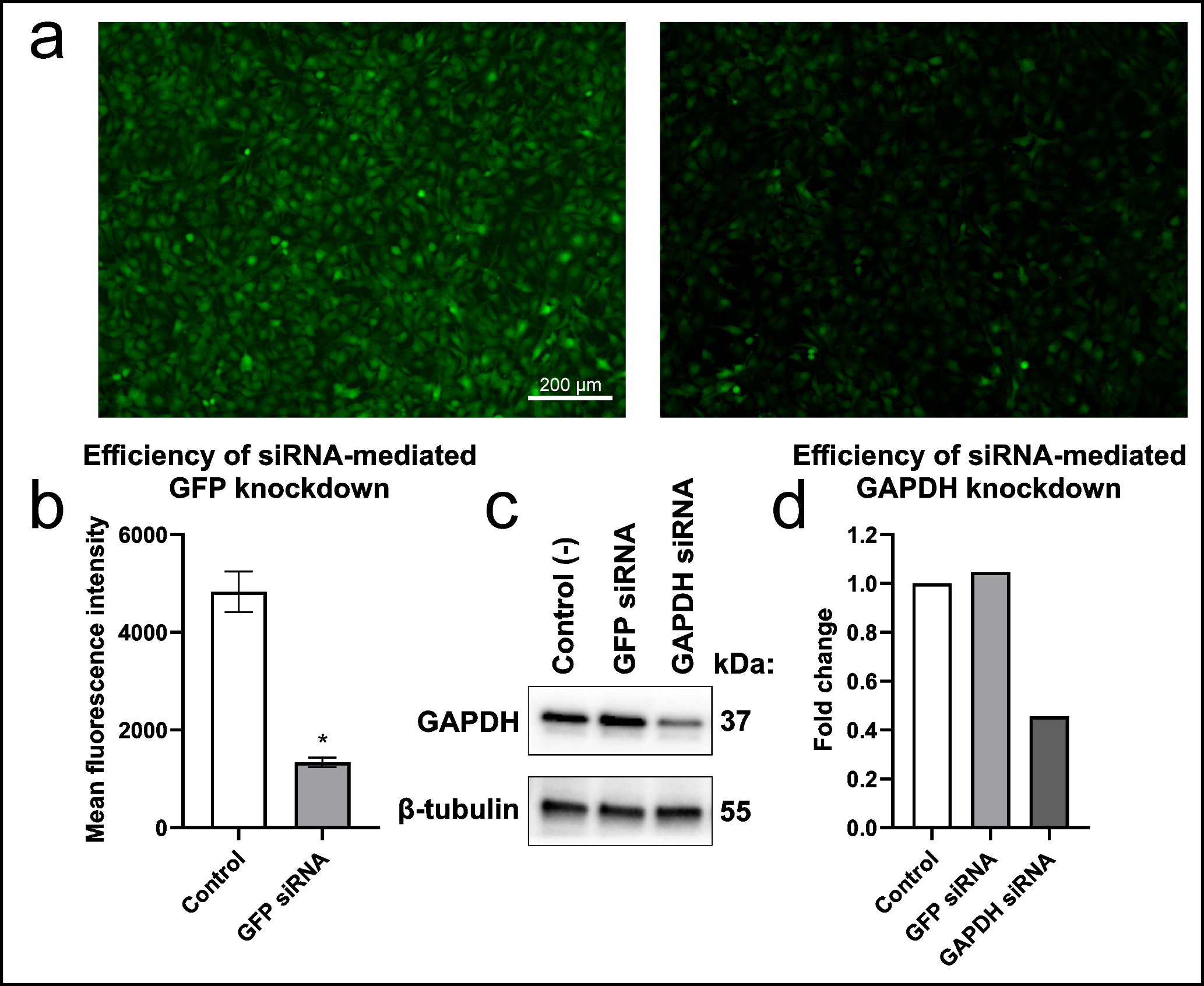


**Fig. S1 Validation of siRNA-mediated knockdown of GFP and GAPDH in 3T3 fibroblasts (a)** representative fluorescence microscopy images of 3T3 fibroblasts stably expressing GFP (control) and the same cells transfected with GFP-targeting siRNA. The scale bar applies to both images. **(b)** Quantification of mean GFP fluorescence (± SEM), based on six fields of view from two independent experiments, demonstrating a marked reduction in GFP signal after siRNA transfection. *Statistically significant difference compared to control (p < 0.05).
**(c)** Western blot detecting GAPDH in control and siRNA-treated cells, with β-tubulin used as the loading control. GFP-targeting siRNA served as a non-targeting control in the GAPDH knockdown experiment. **(d)** Densitometric analysis of GAPDH band intensities normalized to the control.


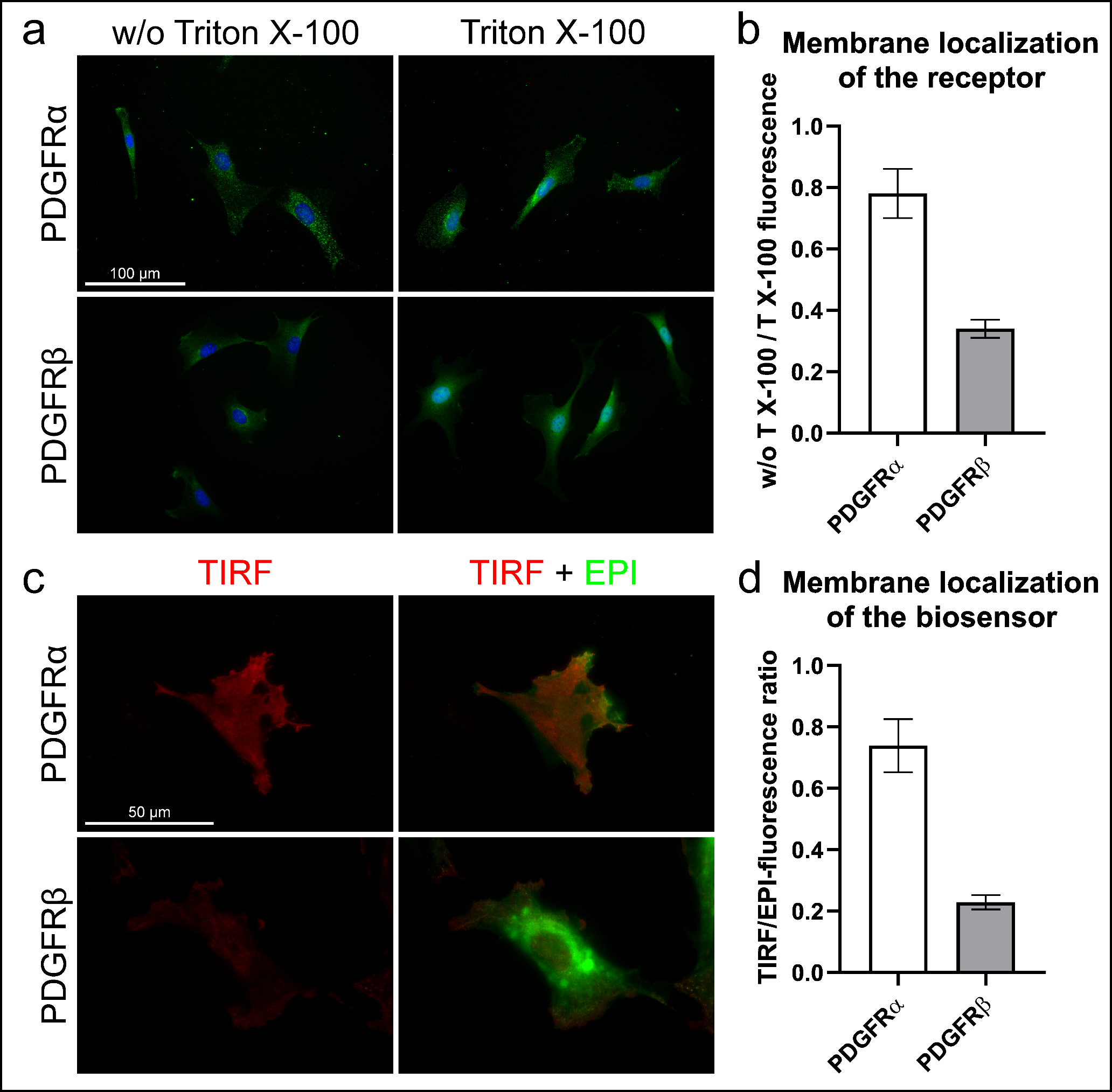


**Fig. S2 Differential membrane localization of PDGFRα and PDGFRβ assessed by immunofluorescence and TIRF microscopy. (a)** Representative immunofluorescence images of PDGFRα and PDGFRβ localization in fibroblasts under non-permeabilized (w/o Triton X-100) and permeabilized (Triton X-100) conditions. Receptors are green while nuclei are blue. Scale bar is common for all images within a panel. **(b)** Quantification of membrane localization of the receptors, calculated as the ratio of fluorescence intensity in non-permeabilized vs. permeabilized conditions presented as mean (± SEM)**.** At least 50 cells were analyzed per condition.
**(c)** Representative TIRF (red) and TIRF+EPI fluorescence (red/green) images of cells expressing PDGFRα-GFP and PDGFRβ-GFP biosensors. Scale bar is common for all images within a panel. **(d)** Quantification of TIRF/EPI fluorescence ratio, presented as mean (± SEM) for n = 13 (PDGFRα) or 11 (PDGFRβ) cells.


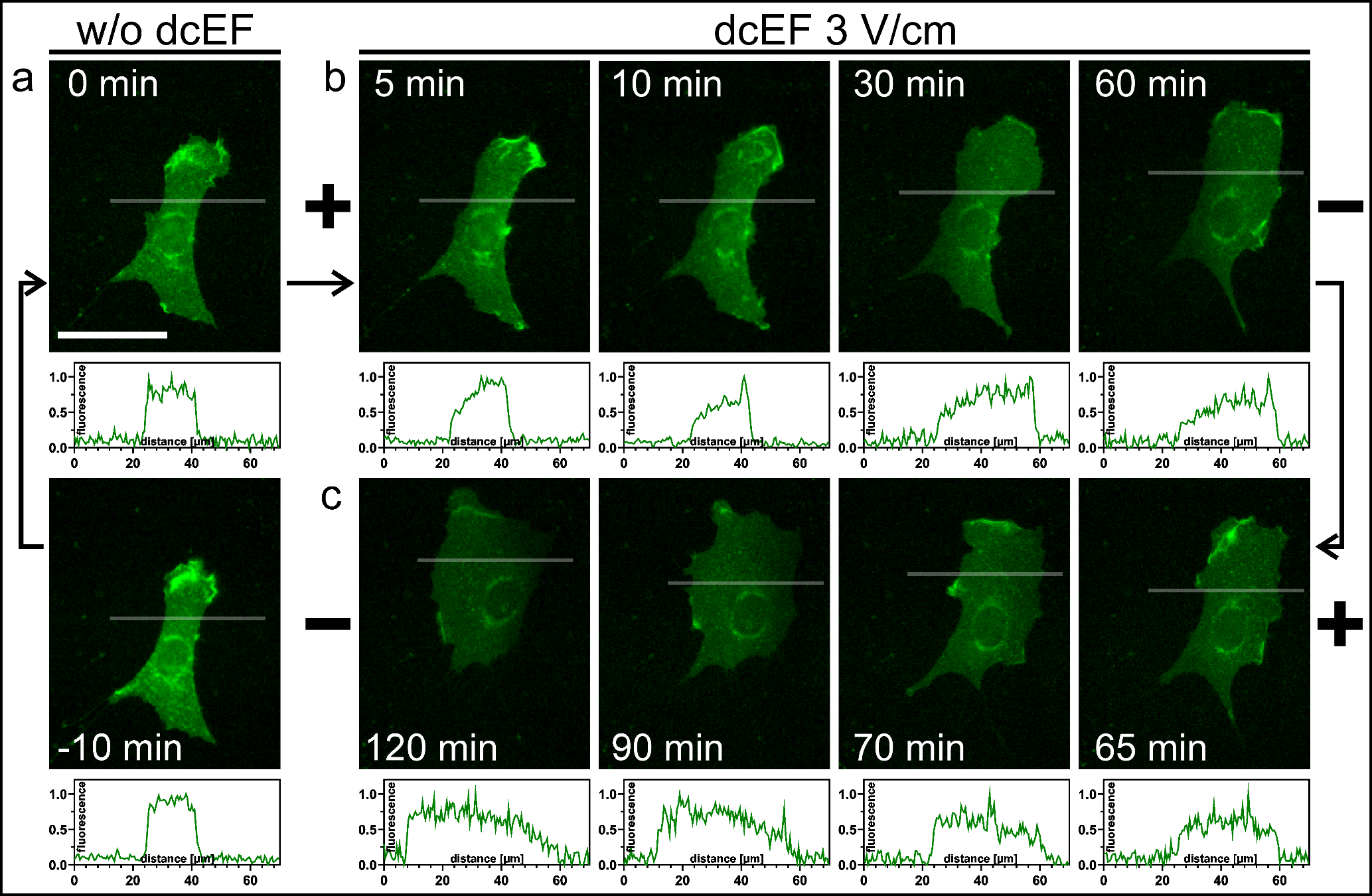


**Fig. S3** **Visualization of PDGF receptor α redistribution following 3 V/cm dcEF application and reversal.** 3T3 fibroblasts transfected with plasmid encoding PDGFRα-GFP were observed using fluorescence microscopy before and during the application of a dcEF (3 V/cm). Initially, a 10 -minute recording captured cells under isotropic conditions **(a)**. Subsequently, the dcEF was applied with the cathode placed on the right side for 60 minutes **(b)**. Following this, the dcEF polarity was reversed by replacing the electrodes and placing the cathode on the left side **(c)**. The line profiles illustrate the relative distribution of fluorescence within the cell, normalized to the brightest pixel along the line. A scale bar of 50 μm is applicable to all images within the figure.


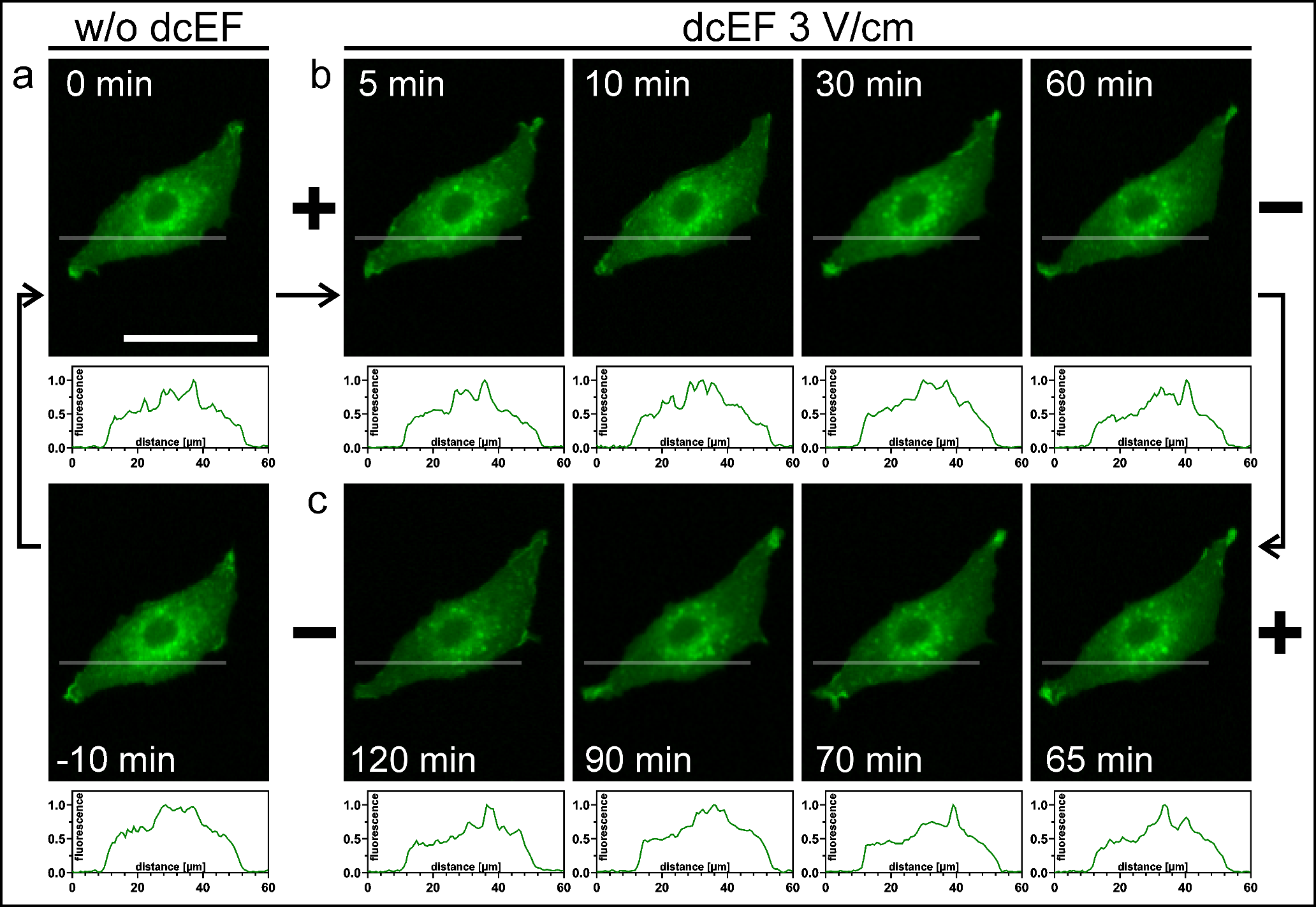


**Fig. S4 Visualization of PDGF receptor β redistribution following 3 V/cm dcEF application and reversal.** 3T3 fibroblasts transfected with plasmid encoding PDGFRβ-GFP were observed using fluorescence microscopy before and during the application of a dcEF (3 V/cm). Initially, a 10 -minute recording captured cells under isotropic conditions **(a)**. Subsequently, the dcEF was applied with the cathode placed on the right side for 60 minutes **(b)**. Following this, the dcEF polarity was reversed by replacing the electrodes and placing the cathode on the left side **(c)**. The line profiles illustrate the relative distribution of fluorescence within the cell, normalized to the brightest pixel along the line. A scale bar of 50 μm is applicable to all images within the figure.

**
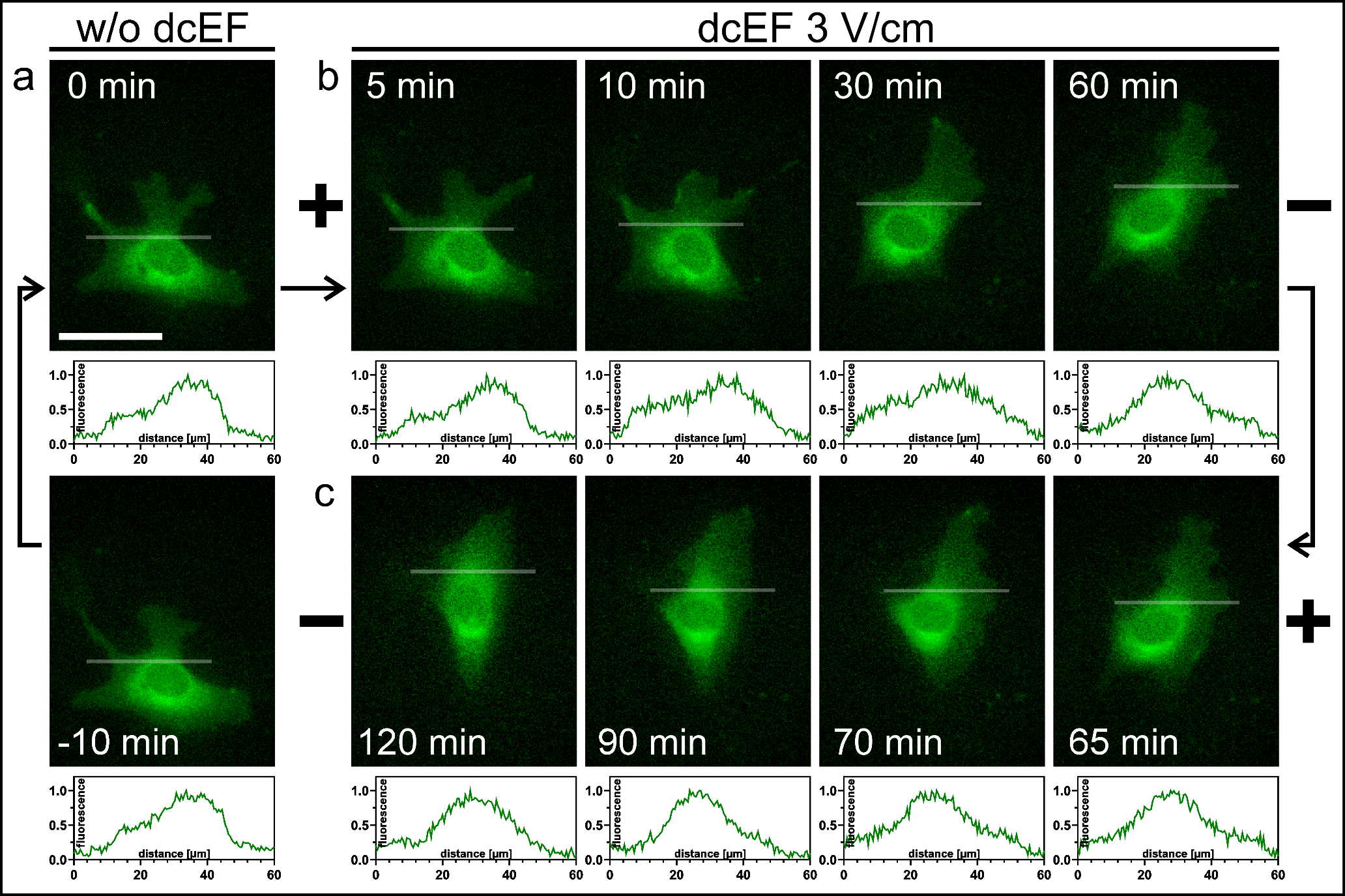
**

**Fig. S5 Visualization of TGFβ-R1-GFP distribution in 3T3 fibroblasts before and during the application of a dcEF (3 V/cm).** Cells transfected with a plasmid encoding TGFβ-R1-GFP were observed using fluorescence microscopy. Initially, a 10 -minute recording captured cells under isotropic conditions **(a)**. Subsequently, the dcEF of 3 V/cm was applied with the cathode placed on the right side for 60 minutes **(b)**. Following this, the dcEF polarity was reversed by replacing the electrodes and placing the cathode on the left **(c)**. The line profiles illustrate the relative distribution of fluorescence within the cell, normalized to the brightest pixel along the line. In order to minimize noise level, measured signal was averaged from five adjacent parallel lines, each of 1 px. thickness. A scale bar of 50 μm is applicable to all images within the figure.


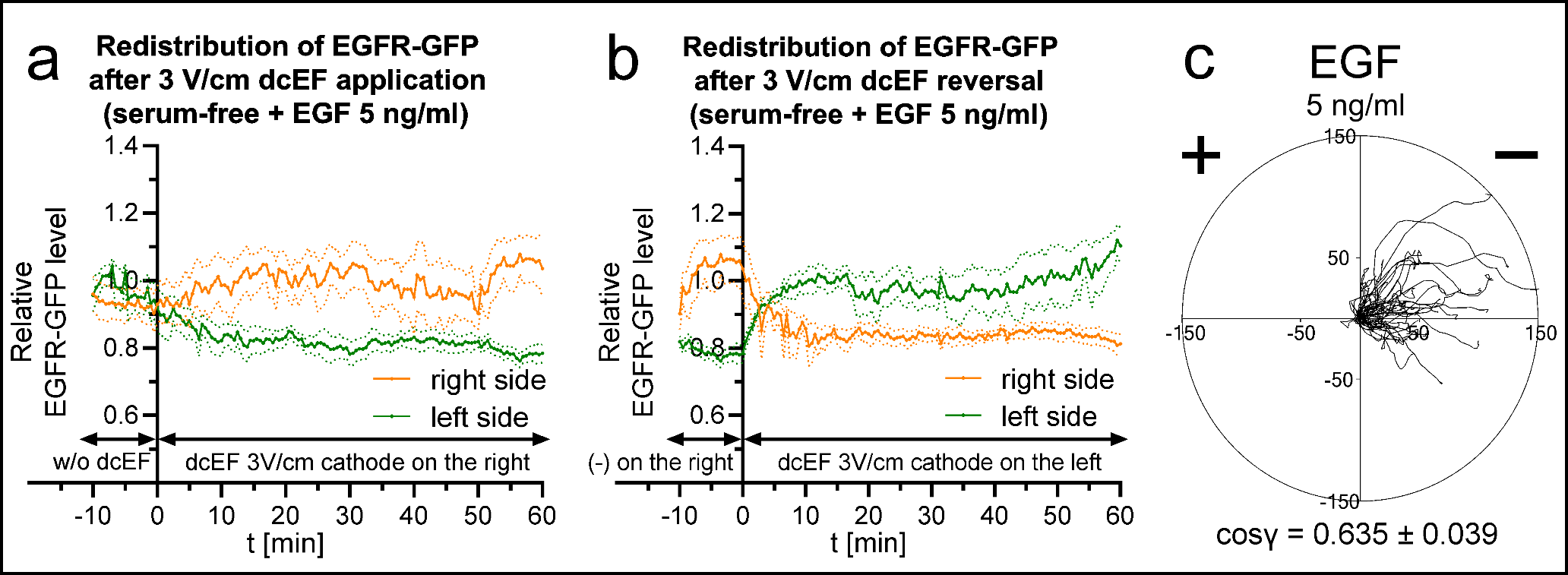


**Fig. S6 The influence of EGF (5 ng/ml) on the redistribution of EGFR-GFP, long term electrotaxis of 3T3 fibroblasts (a)** - redistribution of the EGFR-GFP in the presence of EGF (5 ng/ml) after the application of dcEF (3 V/cm) at the 0-minute time point, with the cathode placed on the right side of the field of view.
**(b)** - redistribution of the EGFR-GFP in the presence of EGF (5 ng/ml) following the reversal of the dcEF (3 V/cm) polarity at the 0-minute time point. The graphs **(a, b)** were constructed as previously (Fig. 11), and represent average values (± SEM) for n = 5 cells; **(c)** - Circular diagram showing composite trajectories of individual cell migration (n = 50 cells) under a dcEF of 3 V/cm in serum-free medium supplemented with EGF (5 ng/ml). The initial point of each trajectory (constructed from the subsequent 36 cell centroid positions, recorded at 5-minute intervals) was set at the beginning of the coordinate system. The cathode of the dcEF is located on the right side of the diagram. The scale is in μm.
